# Synthesis and biological activity of α-glucosyl C24:0 and C20:2 ceramides

**DOI:** 10.1016/j.bmcl.2010.05.010

**Published:** 2010-06-15

**Authors:** Peter J. Jervis, Natacha Veerapen, Gabriel Bricard, Liam R. Cox, Steven A. Porcelli, Gurdyal S. Besra

**Affiliations:** a School of Biosciences, University of Birmingham, Edgbaston, Birmingham B15 2TT, UK; b School of Chemistry, University of Birmingham, Edgbaston, Birmingham B15 2TT, UK; c Department of Microbiology and Immunology, Albert Einstein College of Medicine, Yeshiva University, Bronx, NY 10461, USA

**Keywords:** CD1d, *i*NKT, Antigen, Ceramide, Lipid

## Abstract

α-Glucosyl ceramides **4** and **5** have been synthesised and evaluated for their ability to stimulate the activation and expansion of human *i*NKT cells. The key challenge in the synthesis of both target molecules was the stereoselective synthesis of the α-glycosidic linkage. Of the methods examined, glycosylation using per-TMS-protected glucosyl iodide **16** was completely α-selective and provided gram quantities of amine **11**, from which α-glucosyl ceramides **4** and **5** were obtained by N-acylation. α-GlcCer **4**, containing a C24 saturated acyl chain, stimulated a marked proliferation and expansion of human circulating *i*NKT cells in short-term cultures. α-GlcCer **5**, which contains a C20 11,14-*cis*-diene acyl chain (C20:2), induced extremely similar levels of *i*NKT cell activation and expansion.

## Graphical abstract

α-Glucosyl ceramides **4** and **5** have been synthesised and their ability to stimulate the activation and expansion of *i*NKT cells evaluated.

**Figure:**
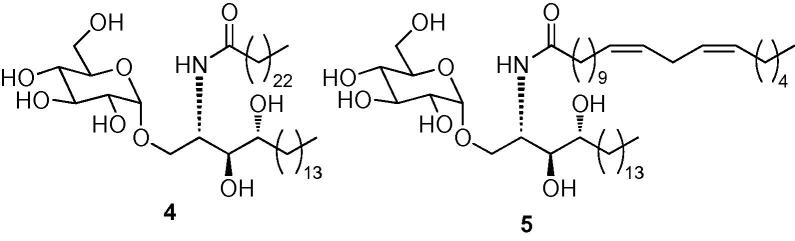

CD1d is a non-polymorphic glycoprotein expressed on the surface of antigen-presenting cells (APCs). It is specifically associated with presenting lipid antigens that activate the distinctive class of T cells known as invariant Natural Killer T (*i*NKT) cells. *i*NKT cells display characteristics of both T cells and NK cells and play a crucial role in diverse immune responses and other pathologic conditions.^1^, ^2^, ^3^, ^4^ When the synthetic glycolipid α-galactosyl ceramide (α-GalCer),^5^ also known as KRN7000 (**1**, Fig. 1), is bound to CD1d and presented to T cell receptors (TCRs) on the surface of *i*NKT cells, the latter are activated to release diverse cytokines, including both Th1 and Th2 cytokines.^6^, ^7^, ^8^ Similar results are obtained with the more readily obtained C24:0 analogue (**2**, Fig. 1).^9^, ^10^ It is believed that the release of Th1 cytokines may contribute to antitumour and antimicrobial functions, whilst the secretion of Th2 cytokines may help alleviate autoimmune diseases^11^, ^12^, ^13^ such as multiple sclerosis^14^ and arthritis.^15^ The opposing effects induced by Th1 and Th2 cytokines have complicated efforts to develop KRN7000 as a therapeutic agent, since it induces high levels of both types of cytokine and therefore may induce mixed and unpredictable biological effects.^16^ Switching the C26:0 acyl chain of KRN7000 for a C20 11,14-*cis*-diene acyl chain modifies the outcome of *i*NKT cell activation and potently induces a Th2-biased cytokine response.^9^ This C20:2 analogue (**3**, Fig. 1) also exhibits less stringent requirements for loading on to CD1d.^10^

**Figure 1:**
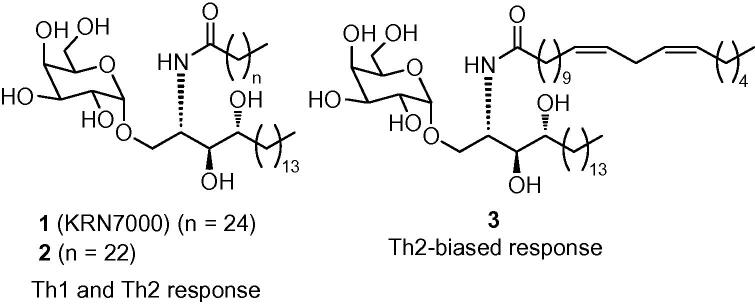
α-Galactosyl ceramides **1** (C26:0), **2** (C24:0) and **3** (C20:2).

Although extensive studies have examined the impact on the *i*NKT cell-stimulating activities of modifications to the fatty acyl and sphingosine structures of α-GalCer, there has been less analysis of the effects of structural modifications of the carbohydrate head group.^17^ Subtle changes in this part of the glycolipid are likely to have significant effects on *i*NKT cell recognition since the monosaccharide group is exposed and makes direct contacts with the TCR in complexes formed by the binding of α-GalCer to CD1d.^18^ To this end, we now report the synthesis and preliminary biological activity of α-glucosyl ceramide analogues **4** and **5** (Fig. 2).

**Figure 2:**
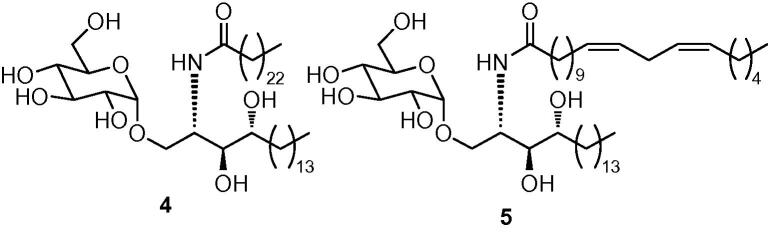
Target α-glucosyl ceramides **4** (C24) and **5** (C20:2).

Since targets **4** and **5** differ only in their acyl chain substitution, we elected to pursue a synthetic strategy that would allow the introduction of this point of diversity in the final step. We therefore examined several routes to amine **11** from which both glucosyl ceramide targets would then be accessed through chemoselective acylation of the amino residue. The key challenge in a synthesis of amine **11** is to form the glycosidic linkage with high α-selectivity. To this end, we first opted to employ a stereospecific glucosylation method developed by Bols (Scheme 1).^19^ This method involves the use of a silyl tether to attach the acceptor temporarily to the 2-position of the glucosyl donor prior to the key glycosylation step. Glycosylation proceeds with 1,2-*syn* specificity, owing to the formation of a five-membered silylacetal intermediate, which in the case of glucosyl donors, ensures the formation of the α-glycoside product. Thioglucoside **7**, synthesised in three steps from d-glucal **6**,^20^ was reacted with a fivefold excess of dichlorodimethylsilane. This reaction afforded a silyl chloride intermediate, which, after removal of the excess dichlorosilane reagent under reduced pressure, reacted with known alcohol **8**^21^ to form mixed silyl acetal **9**, our glucosylation precursor, in modest yield. Treatment of silyl acetal **9** with *N*-iodosuccinimide (NIS) furnished the desired glucoside **10** as a single diastereoisomer, albeit in modest yield. Hydrogenolysis of the benzyl groups and reduction of the azide in **10** using Pd(OH)_2_ as the catalyst,^22^ provided our acylation precursor, amine **11** in 57% yield (Scheme 1).

**Scheme 1:**
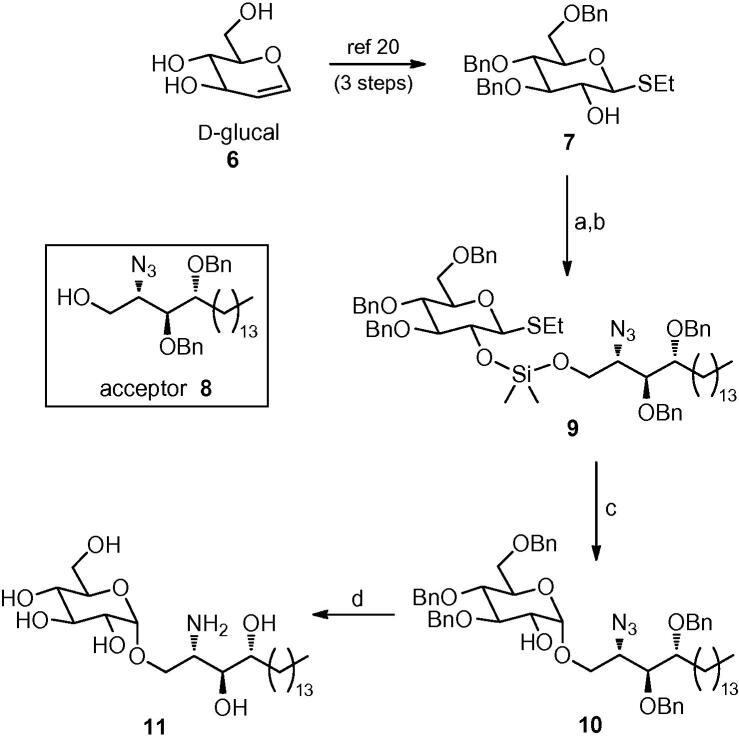
Reagents: (a) Me_2_SiCl_2_, pyridine, toluene; (b) acceptor **8**, pyridine, toluene, 38% over two steps; (c) NIS, MeNO_2_, 47%; (d) H_2_, Pd(OH)_2_, CHCl_3_/MeOH (1:1), 57%.

Although this synthetic approach allowed a completely stereoselective route to our target amine **11**, a number of steps in the sequence suffered from poor yields, which hindered access to significant quantities of material. We therefore examined other glycosylation methods. Kobayashi has described a stereoselective α-glycosylation using a galactosyl bromide generated in situ from 2,3,4,6-tetra-*O*-benzyl galactose.^23^ Unfortunately, we found that glycosylation using the corresponding glucosyl bromide derived from **12** afforded significant amounts of the unwanted β-anomer (Scheme 2). The use of perbenzylated glucosyl fluoride **13**^24^ also provided a mixture of α- and β-glycosides **14**, which proved difficult to separate (Scheme 2).

**Scheme 2:**
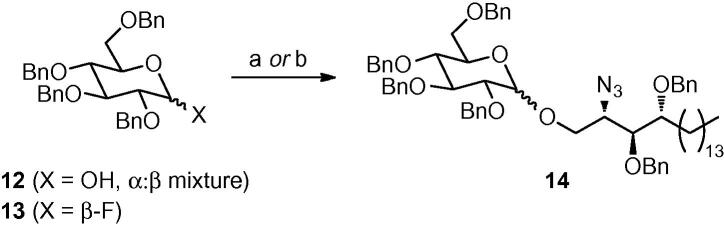
Reagents: (a) From **12**: CBr_4_, PPh_3_, CH_2_Cl_2_, then **8**, *^n^*Bu_4_NBr, tetramethyl urea, 67% (α:β ratio: 1:1); (b) from **13**: **8**, SnCl_4_, AgIO_4_, THF, 41% (α:β ratio: 5:1).

We therefore turned our attention to the use of glucosyl iodides,^25^ specifically per-TMS-protected glucosyl iodide **16**, as an alternative donor. Du et al. have shown that the corresponding galactosyl iodide provides excellent levels of α-selectivity with a variety of alcohol acceptors.^26^ The reaction conditions for this glycosylation are also extremely mild and the silyl protecting groups are easily removed using an acid work-up. We reasoned that the use of phytosphingosine acceptor **17**,^27^ in which the internal 1,2-diol is protected as an acetal, would deliver the completely O-deprotected glucoside **18** upon acid work-up. To this end, 1,2,3,4,6-penta-*O*-trimethylsilyl glucose **15**, which is commercially available or can be readily synthesised on large scale by treating glucose with a mixture of TMSCl and hexamethyldisilazane (HMDS) in pyridine,^28^ was converted to glycosyl iodide **16** by treatment with TMSI in CH_2_Cl_2_ (Scheme 3). Adding a solution of crude **16** to a solution of alcohol **17**, *^n^*Bu_4_NI, Hünig’s base and 4 Å molecular sieves in CH_2_Cl_2_ successfully effected glycosylation. Treating the initially formed glycoside product with *p*-toluenesulfonic acid (*p*TSA) in methanol provided the fully O-deprotected glycoside **18** as a single anomer. Although the yield for this three-step process was a modest 45%, we now had very rapid access to our target molecules. A final Staudinger reduction of azide **18** delivered our requisite amine **11** in quantitative yield (Scheme 3).^29^ This reaction sequence is short and scalable and proved to be particularly effective for accessing multigram quantities of amine **11**. The final acylation reactions were accomplished by adding either tetracosanoyl chloride or 11,14-eicosadienoyl chloride (formed from the corresponding carboxylic acids using oxalyl chloride) in THF to amine **11** in a vigorously stirred biphasic mixture of THF and 8 M NaOAc solution. Both reactions provided the desired amide products **4** and **5** in good yields (Scheme 3).^30^, ^31^

**Scheme 3:**
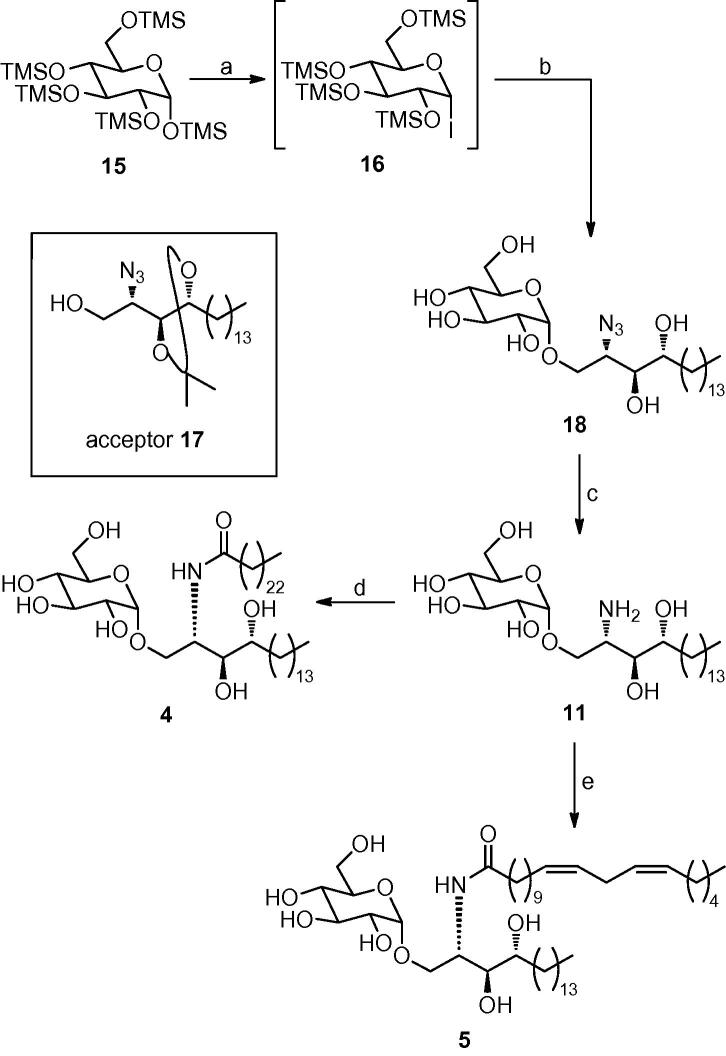
Reagents: (a) TMSI, CH_2_Cl_2_; (b) **17**, *^n^*Bu_4_NI, *^i^*Pr_2_NEt, 4 Å molecular sieves, CH_2_Cl_2_; then *p*TSA, MeOH, 45% from **15**; (c) PMe_3_, wet THF, quant.; (d) tetracosanoyl chloride, THF/8 M NaOAc, 68%; (e) 11,14-eicosadienoyl chloride, THF/8 M NaOAc, 66%.

To assess the biological activity of the α-glucosyl ceramides **4** and **5** and compare these to KRN7000 **1** and the α-galactosyl ceramide analogues **2** (C24:0) and **3** (C20:2), we assessed the ability of each compound to induce the expansion of *i*NKT cells in samples of human peripheral blood mononuclear cells (PBMC) during an eight-day in vitro culture.^32^ The results showed that both the percentages and absolute numbers of *i*NKT cells in the cultures were markedly increased to similar levels by stimulation with both of the α-GlcCer analogues **4** and **5** (Fig. 3). The level of *i*NKT cell expansion, at least with a relatively high concentration of the glycolipids (250 nM), was comparable for both of the *N*-acyl variants of α-GlcCer and very similar to levels obtained with the related α-GalCer analogues (**2** (C24:0) and **3** (C20:2)) and with the prototypical *i*NKT cell activator KRN7000 (**1** (C26:0)). Representative profiles obtained by flow cytometry of cultures from one normal blood donor are shown in Figure 3A. This analysis was carried out with PBMC from four separate donors (Fig. 3B). Although differences were observed for the levels of *i*NKT cell expansion between different donors, all donors responded well to the two α-GlcCer analogues. In all cases, these responses were similar to those generated by the analogous α-GalCer compounds.

**Figure 3:**
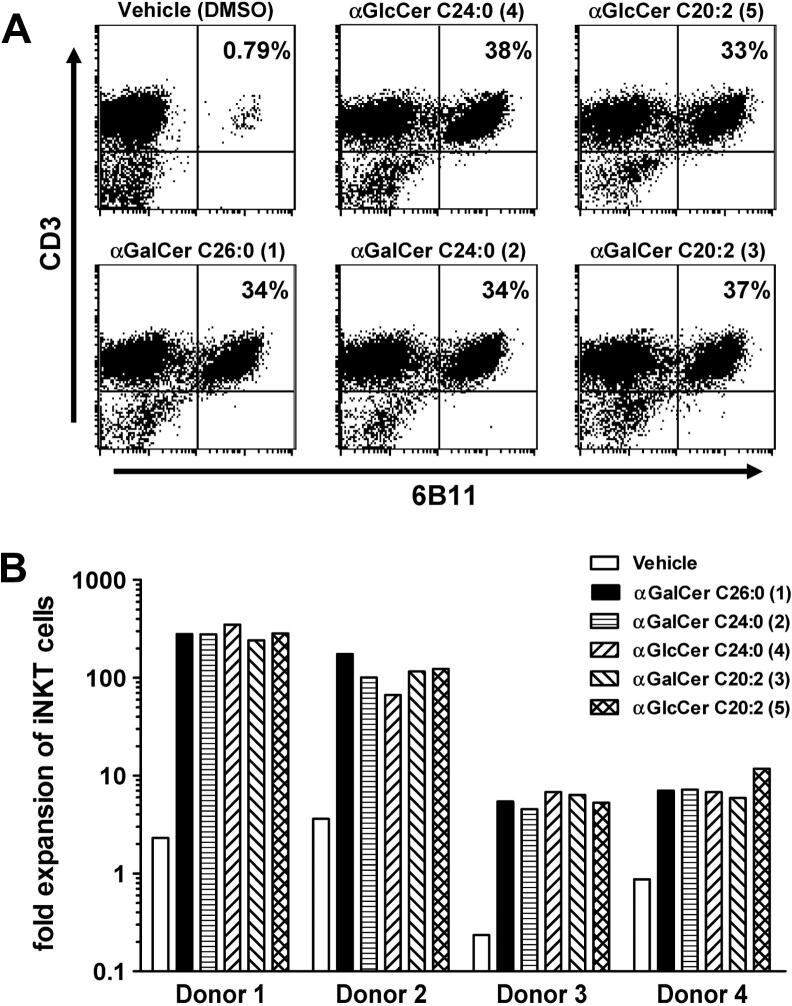
Ex vivo expansion of human *i*NKT cells by α-GlcCer and α-GalCer analogues. Peripheral blood mononuclear cells (PBMC) from four different donors were stimulated with the indicated glycolipids at a concentration of 250 nM in the presence of low levels of exogenous IL-2 and IL-7. At day 8, cultures were harvested and analysed by flow cytometry using monoclonal antibodies specific for CD3 and for the invariant TCRα chain expressed by *i*NKT cells (6B11). (A) Dot plots showing relative levels of CD3^+^ 6B11^+^*i*NKT cells are shown for one representative donor. Numbers in upper right quadrant indicate percentages of total lymphocytes that are *i*NKT cells. (B) Absolute numbers of *i*NKT cells in the cultures were determined by flow cytometry using fluorescent counting beads, and the values of *i*NKT cell fold expansion were determined by dividing by the input number of *i*NKT cells.

The strong biological activity of the α-GlcCer compounds was consistent with findings from the initial study that described the reactivity of CD1d-restricted *i*NKT cells to synthetic glycosylceramides.^5^ This showed an α-GlcCer with a C26 saturated acyl group to be stimulatory for mouse *i*NKT cells, with a level of activity only slightly less than that of KRN7000 (**1**). Our analysis confirms the activity of α-GlcCer compounds as ligands for human *i*NKT cells. It is also notable that we observed human *i*NKT cell activation and expansion for an α-GlcCer with a shorter acyl chain containing unsaturations (**5**). Previous work with analogues of α-GalCer containing C20:2 or other unsaturated fatty acyl groups revealed a marked tendency for these to bias *i*NKT cell-dependent cytokine responses in mice to give preferential secretion of Th2 cytokines such as IL-4 and IL-13.^9^ This Th2 cytokine bias has been associated with therapeutic benefits in a variety of mouse models of autoimmune and inflammatory diseases, indicating potential therapeutic applications for such glycolipids in human diseases.^17^ It will thus be important to determine whether compound **5** or other α-GlcCer analogues bearing an unsaturated acyl chain also show an ability to induce Th2-biased cytokine responses, which is a focus for future studies.

In summary, we have developed an efficient route to α-glucosyl ceramides that provided two biologically active ligands **4** and **5** for stimulation of human *i*NKT cell responses. Of the range of glycosylation methods that were investigated for accessing the target molecules with high levels of stereoselectivity, the use of per-TMS-protected glucosyl iodide **16** as the donor is the most attractive, reacting with acceptor **17** to provide a single α-glycoside product. This glycosylation reaction is also scalable and with an acidic work-up effecting global deprotection, followed by Staudinger reduction of the azide, allows rapid access to advanced intermediate **11**, which can now be used to provide a broad range of α-GlcCer compounds with different acyl chains. Compounds produced using this approach will assist in expanding the current understanding of the structure–activity relationships for glycolipid activators of *i*NKT cells, which is of central importance to the further development of this class of compounds as clinically useful immunomodulators.

## Supplementary data

Supplementary data Experimental procedures.
