# Supplementary material for: Synthesis and biological activity of α-glucosyl C24:0 and C20:2 ceramides
Source: Bioorg Med Chem Lett. 2010 Jun 15;20(12):3475–8. doi: 10.1016/j.bmcl.2010.05.010 (PMC4374101; doi:10.1016/j.bmcl.2010.05.010)
Supplement: Supplementary data — Experimental procedures. [file mmc1.doc]

Synthesis and Biological Activity of α-Glucosyl C_24:0_ and C_20:2_ Ceramides

Peter J. Jervis^a^, Natacha Veerapen^a^, Gabriel Bricard^c^, Liam R. Cox^b^, Steven A. Porcelli^c^, Gurdyal S. Besra^a^*

^a^School of Biosciences, University of Birmingham, Edgbaston, Birmingham, B15 2TT, UK, ^b^School of Chemistry, University of Birmingham, Edgbaston, Birmingham, B15 2TT, UK, ^c^Department of Microbiology and Immunology, Albert Einstein College of Medicine, Yeshiva University, Bronx, NY 10461, USA

* Corresponding author: Tel. +44 0121 41858125; E-mail: g.besra@bham.ac.uk

**Supporting Information**

**Experimental Procedures**

**General Experimental**

Optical rotations were measured using an Optical Activity PolAAr2001 automatic polarimeter. Melting points were determined using open capillaries on a Gallenkamp MPD350 melting point apparatus, and are uncorrected. Infrared spectra were recorded either neat as thin films between NaCl discs on a Perkin Elmer 1600 FTIR spectrometer, or neat on a Perkin Elmer Spectrum 100 fitted with a universal ATR accessory. The intensity of each band is described as s (strong), m (medium) or w (weak), and with the prefix v (very) and suffix br (broad) where appropriate. ^1^H NMR spectra were recorded at 500 MHz, 400 MHz or 300 MHz, using Bruker DRX 500, Bruker AMX 400, Bruker AV 400, Bruker AV 300 and Bruker AC 300 spectrometers. ^13^C NMR spectra were recorded at 125 MHz, 100 MHz or 75 MHz, respectively, using Bruker DRX 500, Bruker AMX 400, Bruker AV 400, Bruker AV 300 and Bruker AC 300 spectrometers. Chemical shifts are reported as δ values (ppm) referenced to the following solvent signals: CHCl_3_, δ_H_ 7.26; CDCl_3_, δ_C_ 77.0; CH_3_OH, δ_H_ 3.34; CD_3_OD, δ_C_ 49.9. The term ‘stack’ is used to describe a region where resonances arising from non-equivalent nuclei are coincident, and multiplet, m, to describe a region where a resonance arises from a single nucleus (or equivalent nuclei) but where coupling constants cannot be readily assigned. Mass spectra were recorded on a Micromass LCT spectrometer utilising electrospray ionisation (and a MeOH mobile phase), and are reported as (*m/z* (%)). HRMS were recorded on a Micromass LCT spectrometer using a lock mass incorporated into the mobile phase. All reagents were obtained from commercial sources, and were used without further purification unless stated otherwise. Anhydrous solvents were purchased from Sigma-Aldrich, UK, stored over 4 Å molecular sieves and under an Ar atmosphere. All solutions are aqueous and saturated unless stated otherwise. Reactions were monitored by TLC using pre-coated aluminium-backed ICN silica plates (60A F254) and visualised by UV detection (at 254 nm) and staining with 5% phosphomolybdic acid in EtOH (MPA spray). Column chromatography was performed on Merck silica gel (particle size 40–63 μm mesh) or Fluka 60 (40–60 μm mesh) silica gel.

**Silyl acetal (9)**

Me_2_SiCl_2_ (0.560 mL, 4.60 mmol) was added to a solution of thioglucoside **7**^1^ (454 mg, 0.920 mmol) in pyridine (1.74 mL) and toluene (7.0 mL) at rt. After stirring for 1 h, the volatiles were removed under reduced pressure. A solution of alcohol **8**^2^ (481 mg, 0.920 mmol) in pyridine (1.75 mL) and toluene (1.75 mL) was then added to the silyl chloride intermediate at rt and the reaction mixture was stirred overnight. The reaction mixture was diluted with CH_2_Cl_2_ (10 mL) and then quenched by the addition of NaHCO_3_ solution (10 mL). The phases were separated and the aqueous phase was extracted with CH_2_Cl_2_ (2 × 10 mL). The combined organic extracts were washed with brine (30 mL), dried (MgSO_4_) and concentrated under reduced pressure. The residue was purified by flash column chromatography (5% EtOAc in hexane) to afford silyl acetal **9** as a colourless oil (316 mg, 38%); R*_f_* = 0.26 (5% EtOAc in hexane); ν_max_(film)/cm^-1^ 2919s, 2850m, 2098s (N_3_), 1462w, 1371w, 1264s, 1220m, 1059m, 1018m, 877w, 737s, 704m; δ_H_(300 MHz, CDCl_3_) 0.21 (3H, s, 1 × SiC*H*_3_), 0.25 (3H, s, 1 × SiC*H*_3_), 0.91 (3H, t, *J* 6.8, CH_2_CH_2_C*H*_3_), 1.18-1.58 (27H, stack, alkyl chain, SCH_2_C*H*_3_), 1.59-1.75 (2H, stack, C*H*_2_), 2.60-2.82 (2H, stack, SC*H*_2_CH_3_), 3.45-3.80 (9H, stack), 3.92 (1H, dd, *J* 10.7, 6.8), 4.16 (1H, d with unresolved fine coupling, *J* 10.2), 4.40 (1H, d, *J* 9.3), 4.47-4.80 (8H, stack), 4.82-4.85 (2H, stack), 7.07-7.16 (2H, stack, Ph*H*), 7.20-7.41 (23H, stack, Ph*H*); δ_C_(75 MHz, CDCl_3_) −2.3 (CH_3_, 1 × Si*C*H_3_), −2.0 (CH_3_, 1 × Si*C*H_3_), [14.1, 15.1 (CH_3_, CH_2_CH_2_*CH_3_*, SCH_2_C*H*_3_)], [22.7, 24.7, 25.5, 29.4, 29.60, 29.62, 29.69, 29.74, 29.8, 31.9 (CH_2_, alkyl chain, SC*H*_2_CH_3_, some overlapping resonances)], 63.6 (CH_2_), 63.9 (CH), 69.1 (CH_2_), 72.0 (CH_2_), 73.4 (CH_2_), 73.6 (CH_2_), 74.6 (CH), 74.9 (CH_2_), 75.2 (CH_2_), 78.3 (CH), 78.8 (CH), 79.2 (CH), 79.6 (CH), 86.1 (CH), 87.0 (CH), [127.1, 127.3, 127.54, 127.55, 127.62, 127.71, 127.73, 127.81, 127.86, 127.88, 128.25, 128.29, 128.31, 128.32, 128.33 (CH, 15 × Ph)], [138.0, 138.20, 138.24, 138.5, 138.7 (quat. C, 5 × *ipso*Ph)]; *m*/*z* (TOF ES^+^) 1096.5 ([M+Na]^+^, 100%); HRMS *m*/*z* (TOF ES^+^) 1096.5917. C_63_H_87_N_3_O_8_SSiNa requires 1096.5881.

**Glucoside (10)**

NIS (158 mg, 0.700 mmol, recrystallised from MeOH) was added to a solution of silyl acetal **9** (300 mg, 0.280 mmol) in MeNO_2_ (10 mL) at rt and the reaction mixture was stirred for 4 h. Hydrochloric acid (1 mL, 1 M solution) was then added and the reaction mixture was stirred for 10 min. The reaction mixture was then diluted with CH_2_Cl_2_ (50 mL) and Na_2_S_2_O_3_ solution (50 mL) and the phases were separated. The organic phase was washed with brine (50 mL), dried (MgSO_4_) and concentrated under reduced pressure. Purification of the residue by flash column chromatography (10% EtOAc in hexane) afforded glucoside **10** (single α-anomer) as a colourless oil (128 mg, 47%); R*_f_* = 0.27 (10% EtOAc in hexane); [α]_D_^20^ +18.9 (c = 0.50 in CHCl_3_); ν_max_(film)/cm^-1^ 3307m (br, OH), 2916s, 2849s, 2095s (N_3_), 1465m, 1380m, 1271m, 1221s, 1166m, 1098s, 1075s, 1032m, 982s, 909s, 881s, 860m, 723s, 685m; δ_H_(500 MHz, CDCl_3_) 0.91 (3H, t, *J* 6.6, C*H*_3_), 1.22-1.44 (24H, stack), 1.50-1.79 (2H, stack), 3.55 (1H, dd, *J* 10.5, 1.2), 3.59-3.80 (8H, stack), 3.80-3.88 (1H, m), 4.16 (1H, dd, *J* 10.4, 2.6), 4.43-4.74 (7H, stack), 4.79-4.96 (4H, stack, including [4.90 (1H, d, *J* 3.0, anomeric-*H*)]), 7.13-7.20 (2H, stack, Ph*H*), 7.23-7.42 (23H, stack, Ph*H*), O*H* not observed; δ_C_(75 MHz, CDCl_3_) 14.1 (CH_3_), [22.7, 25.2, 29.4, 29.59, 29.63, 29.66, 29.67, 29.70, 29.8, 30.0, 31.9 (CH_2_, alkyl chain, some overlapping resonances)], 62.3 (CH), 68.3 (CH_2_), 69.0 (CH_2_), 70.9 (CH), 72.1 (CH_2_), 73.1 (CH), 73.5 (CH_2_), 73.8 (CH_2_), 75.0 (CH_2_), 75.3 (CH_2_), 77.2 (CH), 79.1 (CH), 79.6 (CH), 83.2 (CH), 99.4 (CH, *C*-anomeric), [127.63, 127.66, 127.68, 127.70, 127.80, 127.85, 127.87, 127.99, 128.03, 128.34, 128.36, 128.38, 128.41, 128.43 (CH, 15 × Ph, one overlapping resonance)], [137.8, 137.9, 138.25, 138.27, 138.7 (quat. C, 5 × *ipso*Ph)]; *m*/*z* (TOF ES^+^) 978.5 ([M+Na]^+^, 100%); HRMS *m*/*z* (TOF ES^+^) 978.5620. C_59_H_77_N_3_O_8_Na requires 978.5608.

**1,2,3,4,6-Pentakis-*O*-trimethylsilyl-α-D-glucose (15)**

HMDS (100 mL, 0.480 mol) and TMSCl (50 mL, 0.394 mol) were added sequentially to a solution of D-glucose (10.0 g, 55.5 mmol) in pyridine (500 mL). The solution was stirred at 75 ºC for 1 h under an argon atmosphere before being allowed to cool to rt. The mixture was poured into ice-water (500 mL) and extracted with hexane (3 × 300 mL). The combined organic extracts were washed with water (3 × 300 mL), dried (MgSO_4_) and concentrated under reduced pressure to afford **15** as a viscous, colourless oil (92%); [α]_D_^20^ +62.5 (c = 0.50 in CHCl_3_) (lit.^3^ +65.4 (c = 3.3 in CHCl_3_)); R*_f_* = 0.25 (4% EtOAc in hexane); _max_(film)/cm^-1^ 3608w, 3582w, 3074w, 2957s, 2935s, 2876s, 1780w, 1734w, 1458m, 1415w, 1380m, 1362w, 1342w, 1251s, 1175w, 1131m, 1070s, 1005s, 988s, 945m, 896m, 880s, 841s, 811m, 741s, 726s, 665m, 627m; ™_H_(300 MHz, CDCl_3_) 0.10 (s, 9H, Si(C*H*_3_)_3_), 0.12 (s, 9H, Si(C*H*_3_)_3_), 0.14 (s, 18H, 2 × Si(C*H*_3_)_3_), 0.17 (s, 9H, Si(C*H*_3_)_3_), 3.26-3.42 (2H, m), 3.57-3.80 (4H, m), 4.99 (1H, d, *J* 3.1, *H*-anomeric); δ_C_(75 MHz, CDCl_3_) −0.3 (CH_3_, Si(*C*H_3_)_3_), 0.2 (CH_3_, Si(*C*H_3_)_3_), 0.5 (CH_3_, Si(*C*H_3_)_3_), 1.0 (CH_3_, Si(*C*H_3_)_3_), 1.3 (CH_3_, Si(*C*H_3_)_3_), 62.3 (CH_2_), 72.3 (CH), 72.5 (CH), 74.0 (CH), 74.2 (CH), 93.9 (CH, anomeric-*C*); *m*/*z* (TOF ES^+^) 564.1 ([M+Na]^+^, 100%). Data were in agreement with those reported in the literature.^3,4^

**Azide (18)**

TMSI (274 µL, 2.01 mmol) was added to a solution 1,2,3,4,6-penta-*O*-trimethylsilyl-D-glucose **15** (1.09 g, 2.01 mmol) in CH_2_Cl_2_ (20 mL) at 0 °C. The reaction mixture was stirred under an argon atmosphere for 15 min before benzene (10 mL) was added. The solvent was removed under reduced pressure and the glycosyl iodide intermediate (**16**) obtained was dissolved in CH_2_Cl_2_ (20 mL) and kept under an argon atmosphere. In a separate flask, a mixture of activated 4 Å molecular sieves (500 mg), *^n^*Bu_4_NI (1.48 g, 4.02 mmol), *^i^*Pr_2_NEt (526 µL, 3.02 mmol) and alcohol **17** (257 mg, 0.670 mmol) in CH_2_Cl_2_ (20 mL) was prepared and stirred under an argon atmosphere at rt for 15 min. The solution of glycosyl iodide **16** in CH_2_Cl_2_ was then added dropwise over 5 min to this mixture and the resulting mixture was stirred overnight. After removal of the solvent under reduced pressure, Et_2_O (20 mL) and H_2_O (20 mL) were added and the phases were separated. The organic phase was concentrated under reduced pressure, MeOH (20 mL) and *p*TSA·H_2_O (13.0 mg, 7.00 µmol) were added and the reaction mixture was stirred for 5 h at rt. The reaction was quenched by the addition of NaHCO_3_ (100 mg, 1.19 mmol), filtered and then concentrated under reduced pressure. The resulting yellow solid was purified by flash column chromatography (15% MeOH in CHCl_3_) to afford glycoside **18** (single α-anomer) as a white solid (160 mg, 45%); mp 172-173 °C; R*_f_* = 0.22 (15% MeOH in CHCl_3_); [α]_D_^20^ +15.2 (c = 0.50 in CHCl_3_:MeOH (1:1)) ν_max_(film)/cm^-1^ 3284m br (OH), 2916s, 2847s, 2117s (N_3_), 2096s (N_3_), 1463m, 1247m, 1116w, 1058m, 1043m, 981m, 950w, 929w, 909w, 879w, 723s; δ_H_(400 MHz, CDCl_3_:CD_3_OD (2:1)) 0.80 (3H, t, *J* 7.0, CH_3_), 1.12-1.39 (24H, stack, alkyl chain), 1.41-1.65 (2H, stack, CH_2_), 3.23-3.28 (1H, m), 3.37 (1H, dd, *J* 9.8, 3.8), 3.48-3.54 (1H, m), 3.54-3.71 (6H, stack), 3.75 (1H, dd, *J* 11.6, 2.4), 4.04 (1H, dd, *J* 10.8, 3.6), 4.78 (1H, d, *J* 3.6, anomeric-*H*); δ_C_(100 MHz, CDCl_3_:CD_3_OD (2:1)) 14.3 (CH_3_), [23.2, 26.3, 30.0, 30.3, 30.4, 32.5, 33.3 (CH_2_, alkyl chain, some resonance overlap)], 62.2 (CH_2_), 62.8 (CH), 68.0 (CH_2_), 71.0 (CH), 72.4 (CH), 72.7 (CH), 73.2 (CH), 74.4 (CH), 75.0 (CH), 100.2 (CH, anomeric-*C*); *m*/*z* (TOF ES^+^) 528.4 ([M+Na]^+^, 100%); HRMS *m*/*z* (TOF ES^+^) 528.3263. C_24_H_47_N_3_O_8_Na requires 528.3261.

**Amine (11)**

**From glucoside 10:** Pd(OH)_2_ (a spatula tip) was added to a solution of glucoside **10** (120 mg, 0.130 mmol) in MeOH:CHCl_3_ (5 mL, 1:1) at rt. The reaction vessel was charged with H_2_ gas (3 × vacuum-H_2_ flushes) and the resulting mixture stirred overnight. Filtration through Celite and removal of the solvent under reduced pressure left a residue which was purified by flash column chromatography (40% MeOH in CHCl_3_) to afford amine **11** (35 mg, 57%) as a white powder.

**From azide 18:** PMe_3_ (0.35 mL of a 1.0 M solution in THF, 0.35 mmol) was added to a solution of azide **18** (160 mg, 0.320 mmol) in wet THF (5 mL) at rt and the resulting solution was stirred for 5 h. H_2_O (7.0 µL, 0.35 mmol) was added and the mixture was stirred for 1 h before being concentrated under reduced pressure. The residual H_2_O was removed by azeotropic distillation with toluene (2 × 10 mL). The residue was then subjected to high vacuum at 60 °C for 24 h to remove the Me_3_PO by-product. The amine **11** was obtained as a white powder (152 mg, quant.) and used in the next step without further purification.

mp 211-213 °C; R*_f_* = 0.25 (40% MeOH in CHCl_3_); [α]_D_^20^ 4.7 (c = 0.50 in CHCl_3_); ν_max_(film)/cm^-1^ 3259m br (OH, NH_2_), 2916s, 2849s, 1599w, 1467m, 1321w, 1077s, 1056m, 1028m, 953m, 918m, 848w, 721s, 674m; δ_H_(300 MHz, CD_3_OD) 0.90 (3H, t, *J* 6.8, C*H*_3_), 1.21-1.45 (24H, stack, alkyl chain), 1.50-1.65 (1H, m, C*H*_a_H_b_), 1.70-1.87 (1H, m, CH_a_*H*_b_), 3.12-3.40 (1H, m), 3.24-3.45 (4H, stack), 3.45-3.54 (1H, m), 3.54-3.60 (1H, m), 3.60-3.72 (2H, stack), 3.82 (1H, dd, *J* 11.7, 2.1), 3.99 (1H, dd, *J* 10.1, 3.5), 4.81 (1H, d, *J* 3.6, *H*-anomeric); δ_C_(75 MHz, CD_3_OD) 14.4 (CH_3_), [23.7, 26.6, 30.5, 30.7, 30.77, 30.78, 30.82, 30.9, 33.1, 35.1 (CH_2_, alkyl chain, some overlapping resonances)], 54.3 (CH), 62.7 (CH_2_), 70.1 (CH_2_), 71.8 (CH), 73.7 (CH), 73.8 (CH), 74.1 (CH), 75.2 (CH), 76.5 (CH), 100.8 (CH, anomeric-*C*); *m*/*z* (TOF ES^+^) 502.4 ([M+Na]^+^, 100%), 480.4 (40, [M+H]^+^); HRMS *m*/*z* (TOF ES^+^) 502.3334. C_24_H_49_NO_8_Na requires 502.3356.

**α-GlcCer (4)**

A solution of tetracosanoic acid (70 mg, 0.18 mmol) in (COCl)_2_ (2.0 mL) was stirred at 70 °C for 2 h, after which time, the solution was cooled to rt, and the (COCl)_2_ was removed under a stream of dry argon. The residual volatiles were removed under reduced pressure. The resulting crude acyl chloride was dissolved in dry THF (1.0 mL) and added, with vigorous stirring, to a solution of amine **11** (38 mg, 0.08 mmol) in THF / NaOAc_(aq)_ (8 M) (1:1, 2.0 mL). Vigorous stirring was maintained for 2 h, after which time, the mixture was left to stand and the phases were separated. The aqueous phase was extracted with THF (2 × 2.0 mL), and the combined organic phases were evaporated under reduced pressure. Purification of the residue by column chromatography (gradient from CHCl_3_ to 15% MeOH in CHCl_3_) afforded glucosyl ceramide **4** as a white solid (47 mg, 71%); mp 151-152 °C; R*_f_* = 0.21 (10% MeOH in CHCl_3_); [α]_D_^20^ +47.2 (c = 0.50 in CHCl_3_:MeOH (1:1)); ν_max_(film)/cm^-1^ 3336s br (OH, NH_2_), 2917s, 2850s, 1623m (C=O), 1463m, 1350s, 1143s, 1071m, 1033m, 798s, 718m; δ_H_(500 MHz, CDCl_3_:CD_3_OD (2:1)) 0.88 (6H, t, *J* 6.7, 2 × terminal CH_3_), 1.20-1.44 (64H, stack, alkyl chain), 1.51-1.69 (4H, stack, alkyl chain), 2.19 (2H, app. t, *J* 7.8, C*H*_2_CONH), 3.31-3.37 (1H, m, 4-*H*), 3.45 (1H, dd, *J* 9.8, 3.8, 2-*H*), 3.52-3.59 (3H, stack, 5-*H*, 3’-*H*, 4’-*H*), 3.62 (1H, app. t, *J* 9.5, 3-*H*), 3.65 (2H, stack, 6-*H*_a_, 1’-*H*_a_), 3.79 (1H, dd, *J* 12.0, 2.5, 6-*H*_b_), 3.86 (1H, dd, *J* 10.5, 4.5, 1’-*H*_b_), 4.17 (1H, app. q, *J* 4.5, 2’-*H*), 4.85 (1H, d, *J* 3.5, 1-*H*), CON*H* resonance not observed; δ_C_(125 MHz, CHCl_3_:CD_3_OD (3:1)) 13.5 (CH_3_, 2 × terminal CH_3_), [22.2, 25.5, 28.9, 29.0, 29.2, 29.27, 29.30, 29.4, 31.5, 32.0, 36.0 (CH_2_, alkyl chain, some overlapping resonances)], 50.1 (CH, *C*-2’), 61.2 (CH_2_, *C*-6), 66.8 (CH_2_, *C*-1’), 69.9 (CH, *C*-4), [71.6 (CH), 71.6 (CH, *C*-2), overlapping resonances], 71.8 (CH), 73.5 (CH, *C*-3), 74.2 (CH, *C*-3’), 98.9 (CH, anomeric-*C*), 174.2 (quat. C, *C*ONH); *m*/*z* (TOF ES^+^) 852.8 ([M+Na]^+^, 100%); HRMS *m*/*z* (TOF ES^+^) 852.6912. C_48_H_95_NO_9_Na requires 852.6905.

**α-GlcCer (5)**

(COCl)_2_ (71 µL, 0.84 mmol) was added to a solution of *cis*-11,14-eicosadienoic acid (86 mg, 0.28 mmol) in CH_2_Cl_2_ (1 mL). The resulting solution was allowed to warm to rt over 4 h, before being concentrated under reduced pressure. The resulting crude acyl chloride was dissolved in dry THF (0.50 mL) and added, with vigorous stirring, to a solution of amine **11** (67 mg, 0.140 mmol) in THF / NaOAc_(aq)_ (8 M) (1:1, 2.0 mL). Vigorous stirring was maintained for 2 h, after which time, the mixture was left to stand and the phases were separated. The aqueous phase was extracted with THF (2 × 1.0 mL), and the combined organic phases were evaporated under reduced pressure. Purification of the residue by column chromatography (gradient from CHCl_3_ to 15% MeOH in CHCl_3_) afforded glucosyl ceramide **5** as a white solid (71 mg, 66%); mp 83-85 °C; R*_f_* = 0.23 (10% MeOH in CHCl_3_); [α]_D_^20^ +41.3 (c = 0.50 in CHCl_3_:MeOH (1:1)); ν_max_(film)/cm^-1^ 3300s br (OH), 3009m, 2922s, 2851s, 1697m (C=O), 1633s (C=C), 1540m, 1466m, 1378w, 1282w, 1215w, 1044m, 760m; δ_H_(400 MHz, CDCl_3_:CD_3_OD (2:1)) 0.86 (3H, t, *J* 6.6, terminal C*H*_3_), 0.87 (3H, t, *J* 7.0, terminal C*H*_3_), 1.17-1.43 (42H, stack, alkyl chain), 1.47-1.64 (4H, stack, alkyl chain), 2.00-2.08 (4H, stack, C*H*_2_CH=CHCH_2_CH=CHC*H*_2_), 2.19 (2H, app. t, *J* 7.6, C*H*_2_CONH), 2.75 (2H, app. t, *J* 6.4, CH=CHC*H*_2_CH=CH), 3.31-3.38 (1H, m, 4-*H*), 3.44 (1H, dd, *J* 9.8, 3.7, 2-*H*), 3.51-3.58 (2H, stack, 5-*H*, 4’-*H*), 3.58-3.73 (4H, stack, 1’-*H*_a_, 3’-*H*, 3-*H*, 6-*H*_a_), 3.78 (1H, dd, *J* 11.8, 2.6, 6-*H*_b_), 3.84 (1H, dd, *J* 10.6, 4.2, 1’-*H*_b_), 4.11-4.18 (1H, m, 2’-*H*), 4.84 (1H, d, *J* 3.7, 1-*H*), 5.25-5.40 (4H, stack, 2 × C*H*=C*H*), CON*H* resonance not observed; δ_C_(100 MHz, CDCl_3_) [15.2, 15.3 (*C*H_3_, 2 × terminal CH_3_)], [23.9, 24.0, 26.9, 27.2, 28.5, 28.6, 30.76, 30.81, 30.9, 31.1, 32.9, 33.3, 33.4, 37.7 (CH_2_, alkyl chain, some resonance overlap)], 51.7 (CH, *C*-2’), 62.8 (CH_2_, *C*-6), 68.6 (CH_2_, *C*-1’), 71.5 (CH, *C*-2), 73.26 (CH), 73.32 (CH), 73.6 (CH), 75.1 (CH, *C*-3), 75.6 (CH, *C*-3’), 100.6 (CH, anomeric-*C*), 129.26 (CH, *C*H=CH), 129.30 (CH, *C*H=CH), 131.38 (CH, *C*H=CH), 131.45 (CH, *C*H=CH), 175.8 (quat. C, *C*ONH); *m*/*z* (TOF ES^+^) 792.7 ([M+Na]^+^, 100%); HRMS *m*/*z* (TOF ES^+^) 792.5963. C_44_H_83_NO_9_Na requires 792.5966.

**References**

1. (a) Cheshev, P.; Marra, A.; Dondoni, A. *Carbohydr. Res*. **2006**, *341*, 2714-2716. (b) Seeberger, P. A.; Eckhardt, M.; Gutteridge, C. E.; Danishefsky, S. J. *J. Am. Chem. Soc.* **1997**, *119*, 10064-10072.

2. (a) Kratzer, B.; Mayer, T. G.; Schmidt, R. R. *Eur. J. Org. Chem*. **1998**, 291-298. (b) Xia, C.; Schümann, J.; Emmanuel, R.; Zhang, Y.; Chen, W.; Zhang, W.; De Libero, G.; Wang, P. G. *J. Med. Chem*. **2007**, *50*, 3489-3496.

3. Johnson, D. A. *Carbohydr. Res*. **1992**, *237*, 313-318.

4. Olah, G. A.; Klumpp, D. A. *Synthesis* **1997**, 744-746.

6


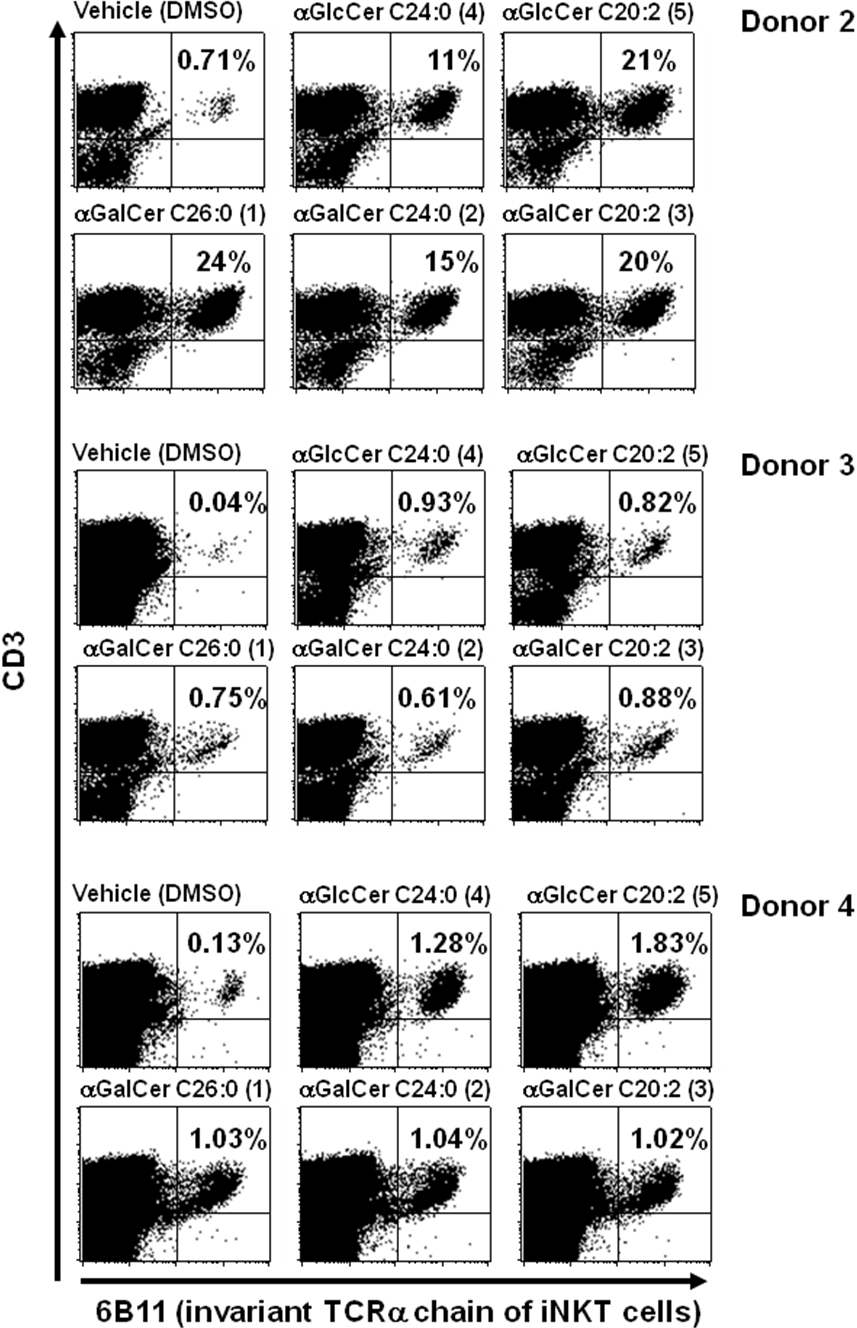


**Supplementary Figure 1. *Ex vivo* expansion of human *i*NKT cells by αGlcCer and αGalCer analogues.** Peripheral blood mononuclear cells (PBMC) from three different donors were stimulated with the indicated glycolipids at a concentration of 250 nM in the presence of low levels of exogenous IL-2 and IL-7. At day 8, cultures were harvested and analysed by flow cytometry using monoclonal antibodies specific for CD3 and for the invariant TCRα chain expressed by *i*NKT cells (6B11). Dot plots showing relative levels of CD3^+^ 6B11^+^ *i*NKT cells are shown for each donor. Numbers in upper right quadrant indicate percentages of total lymphocytes that are *i*NKT cells.


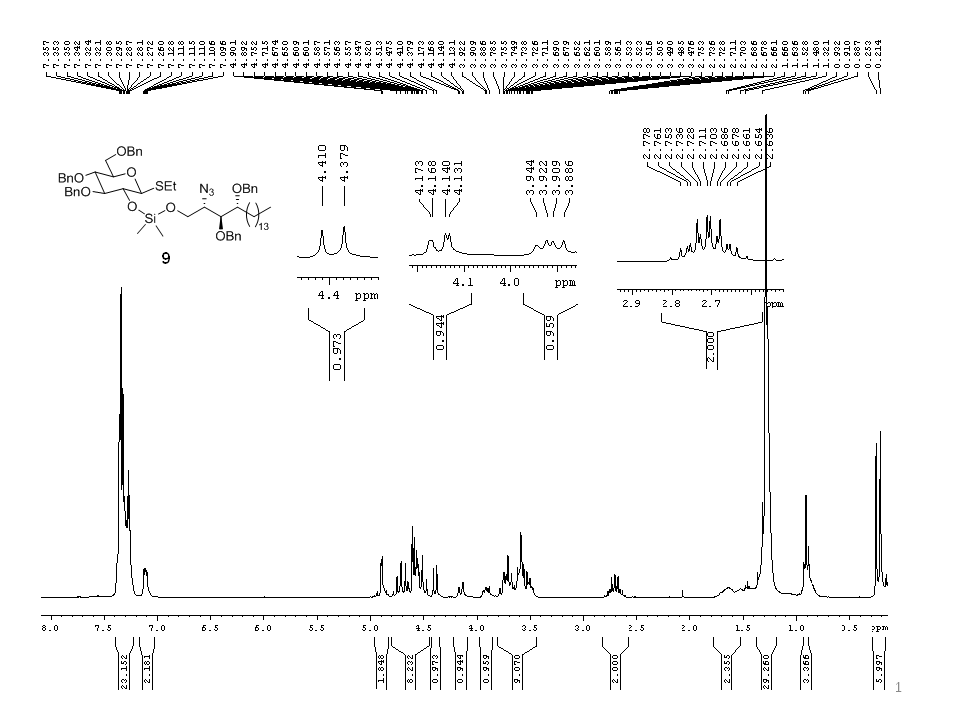

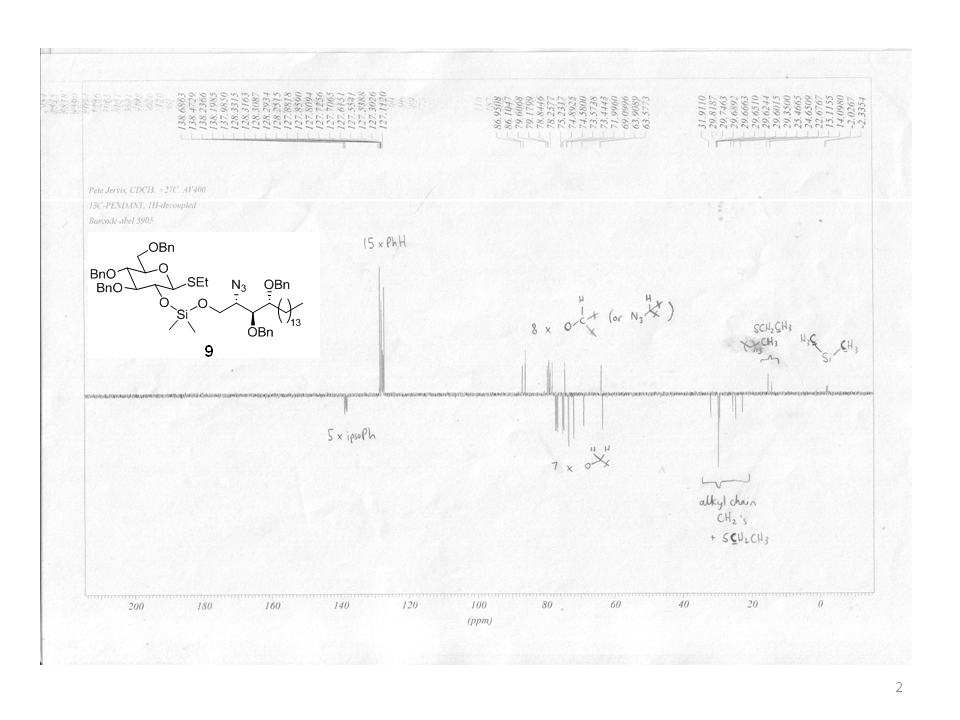

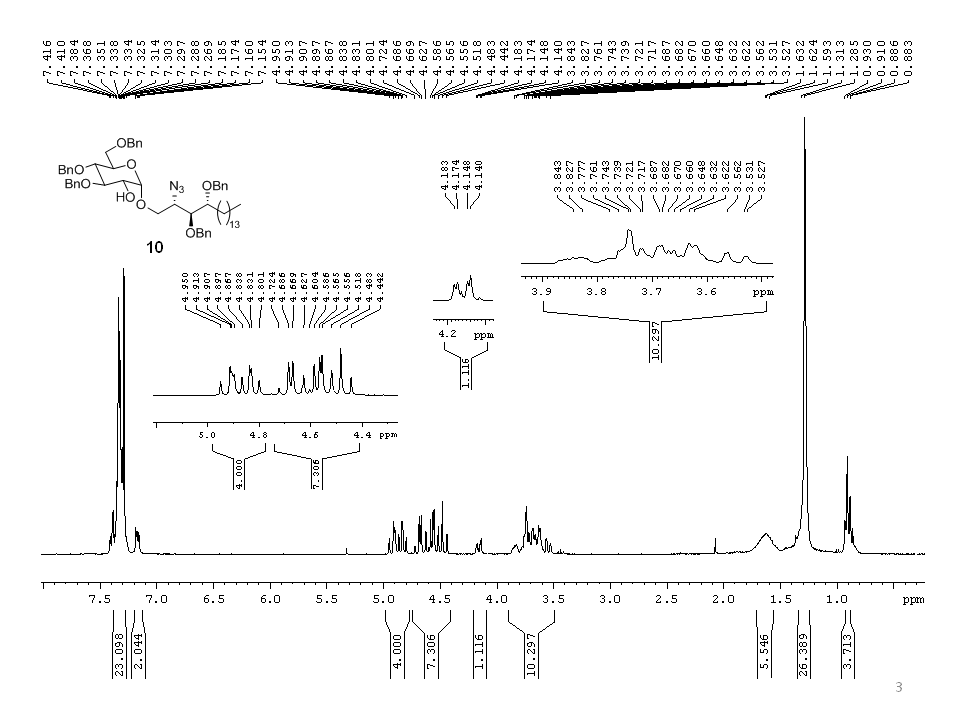


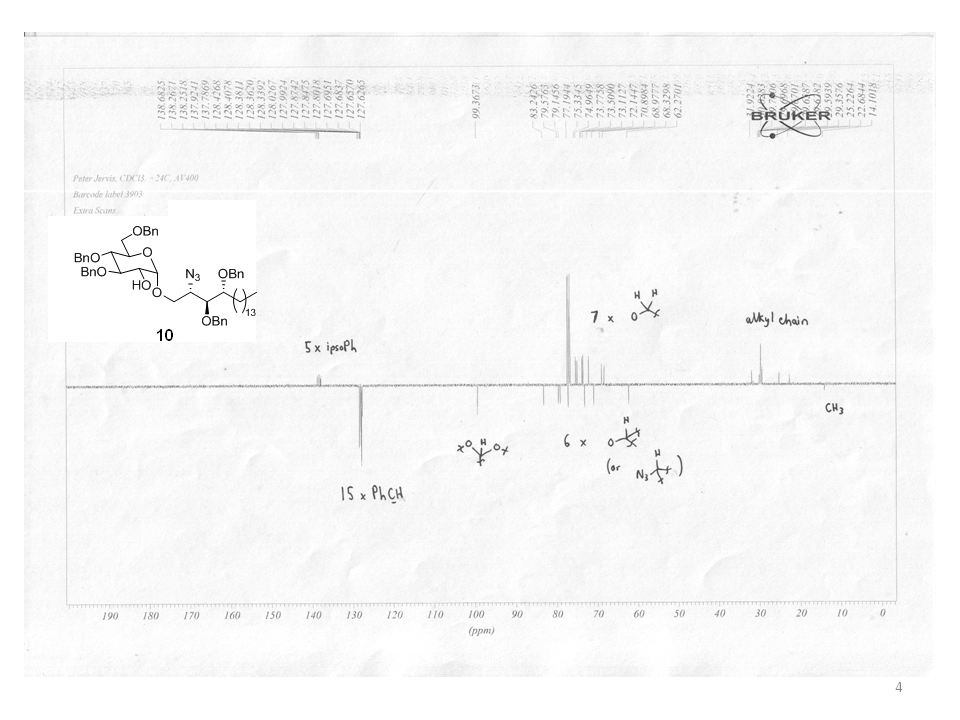


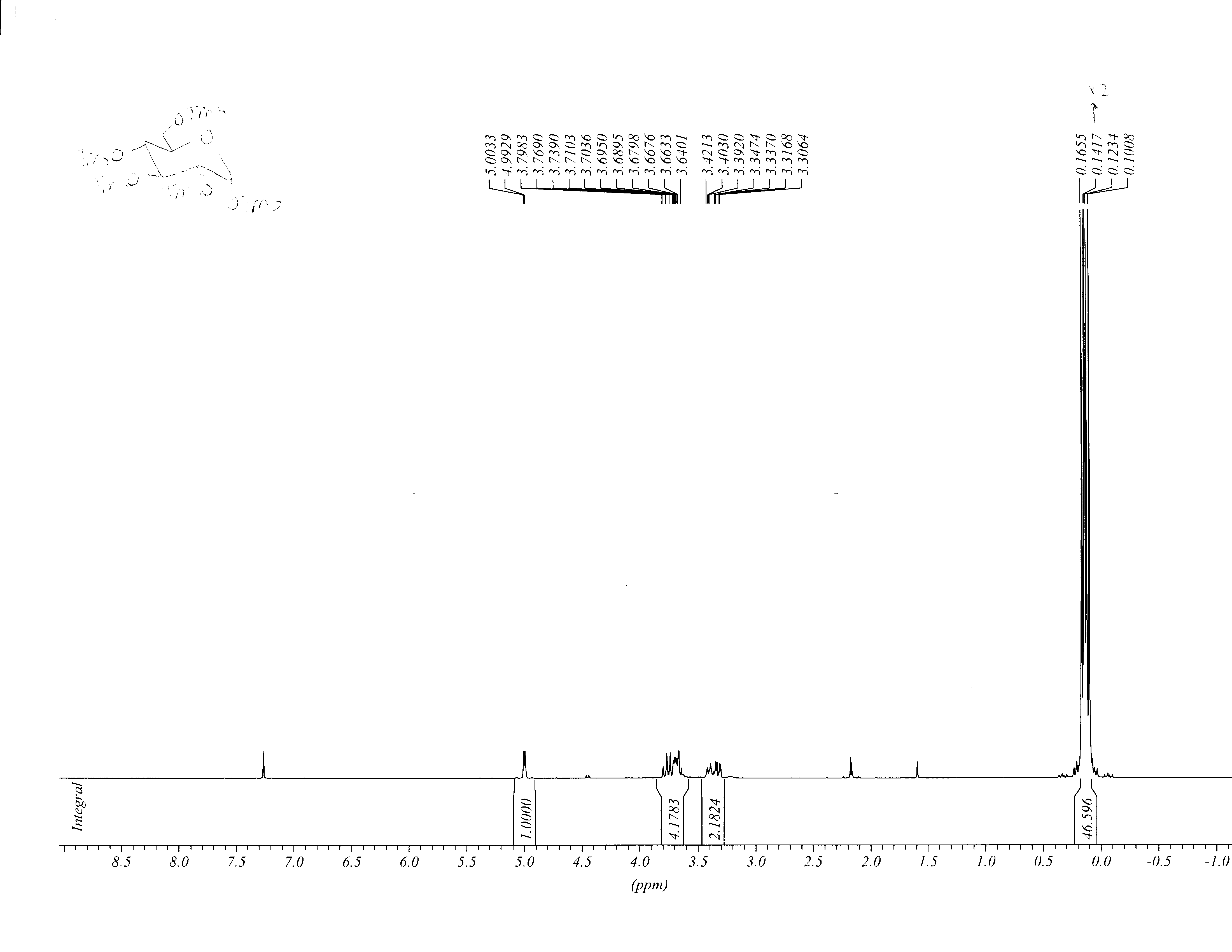


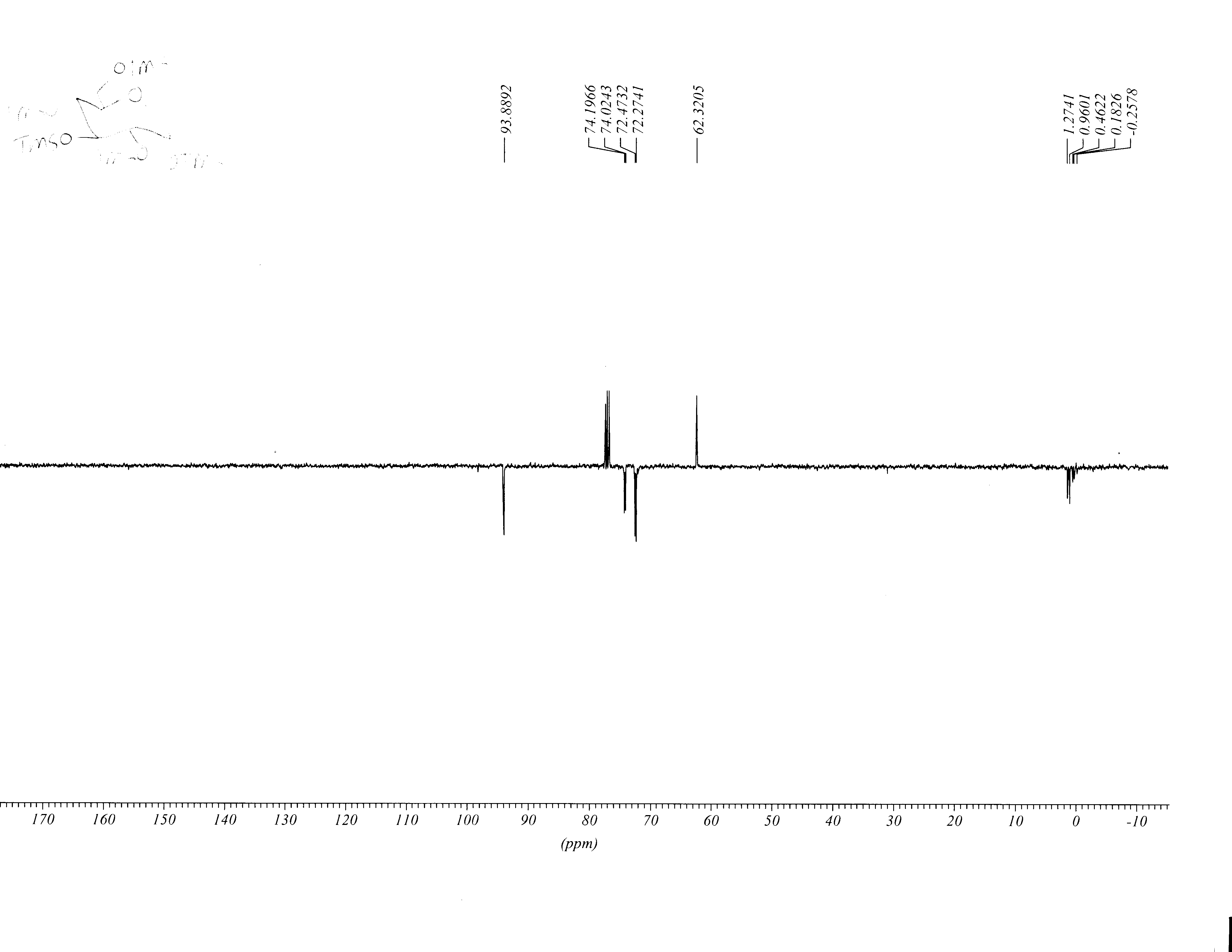


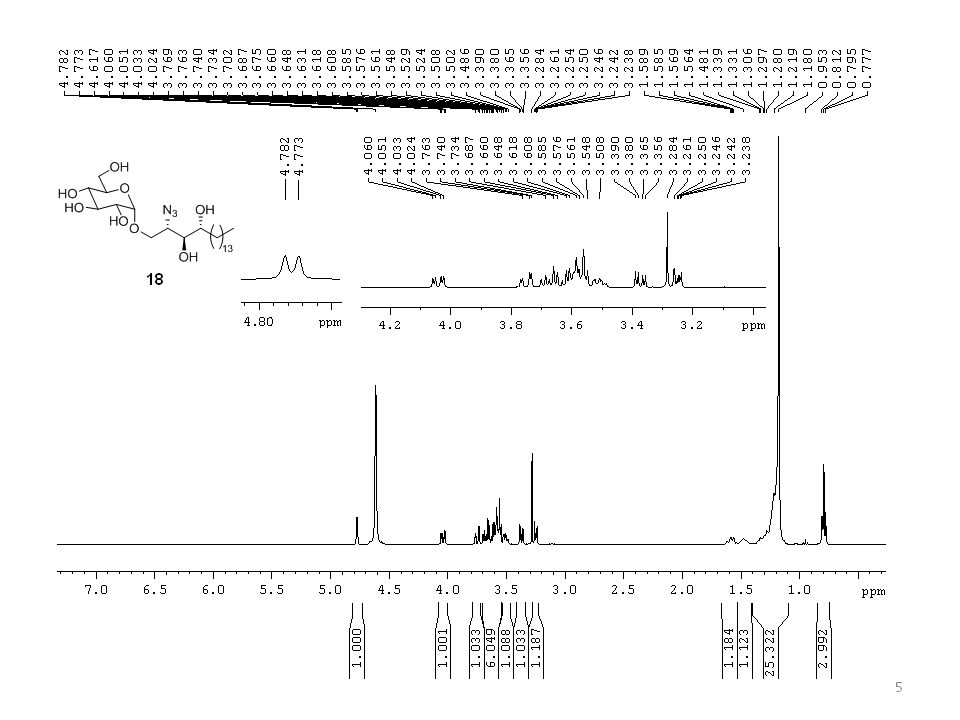


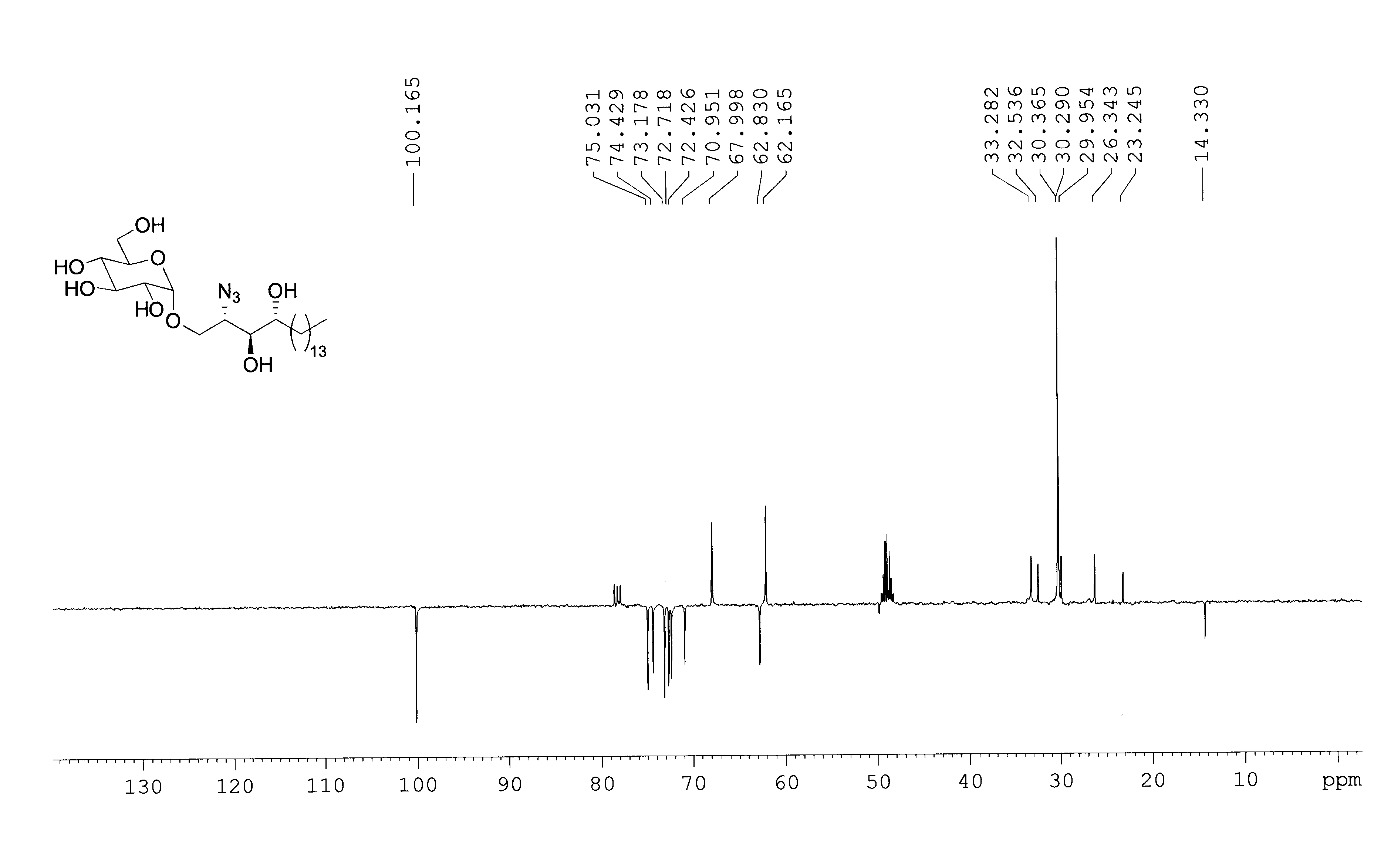

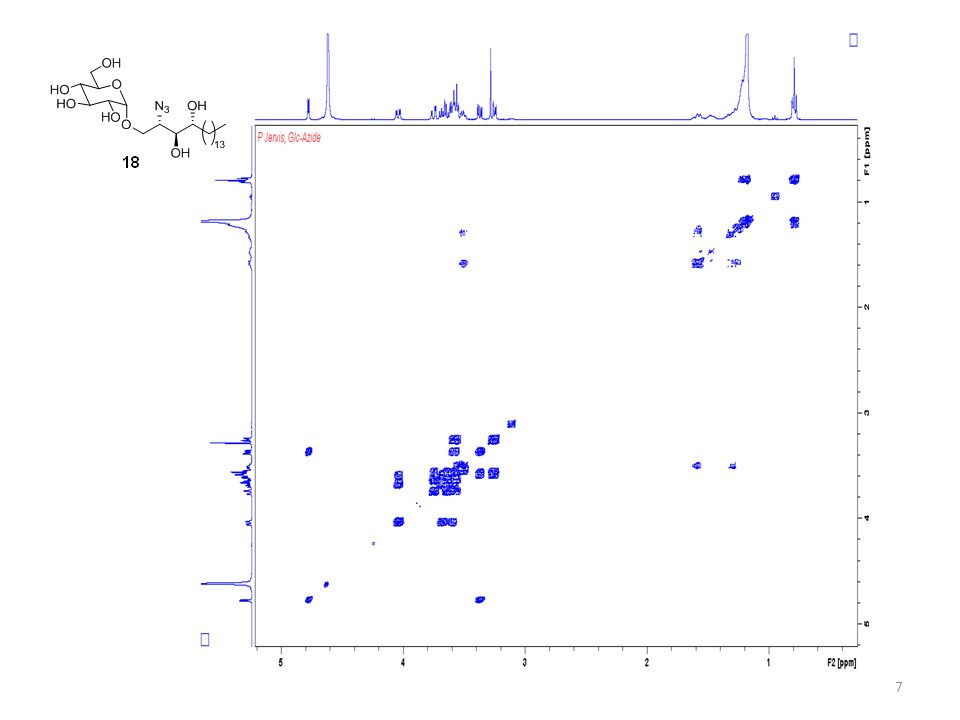

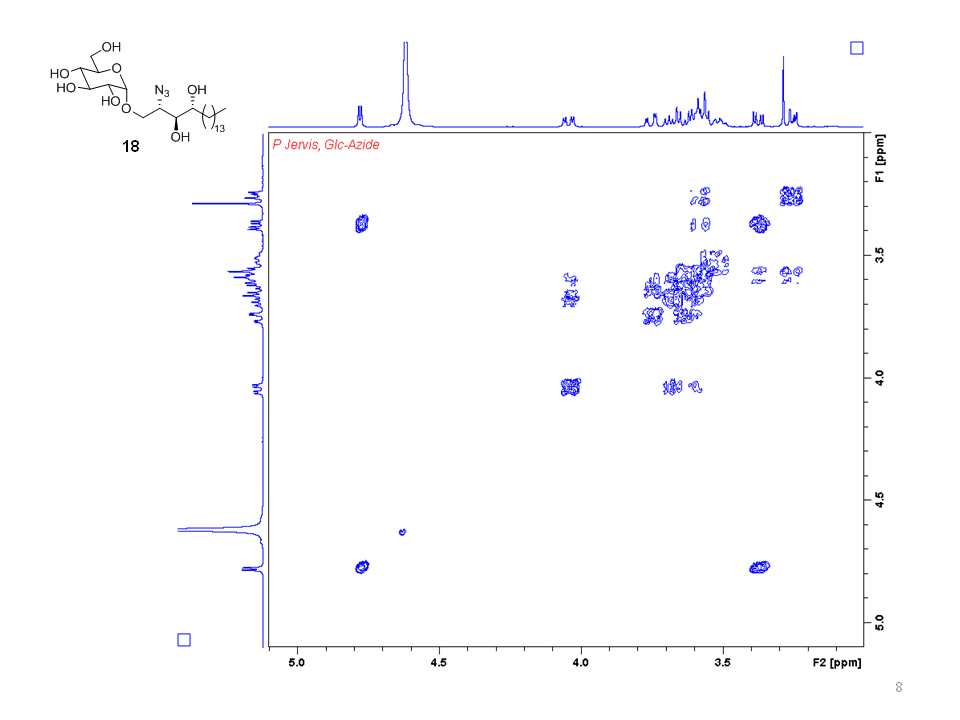

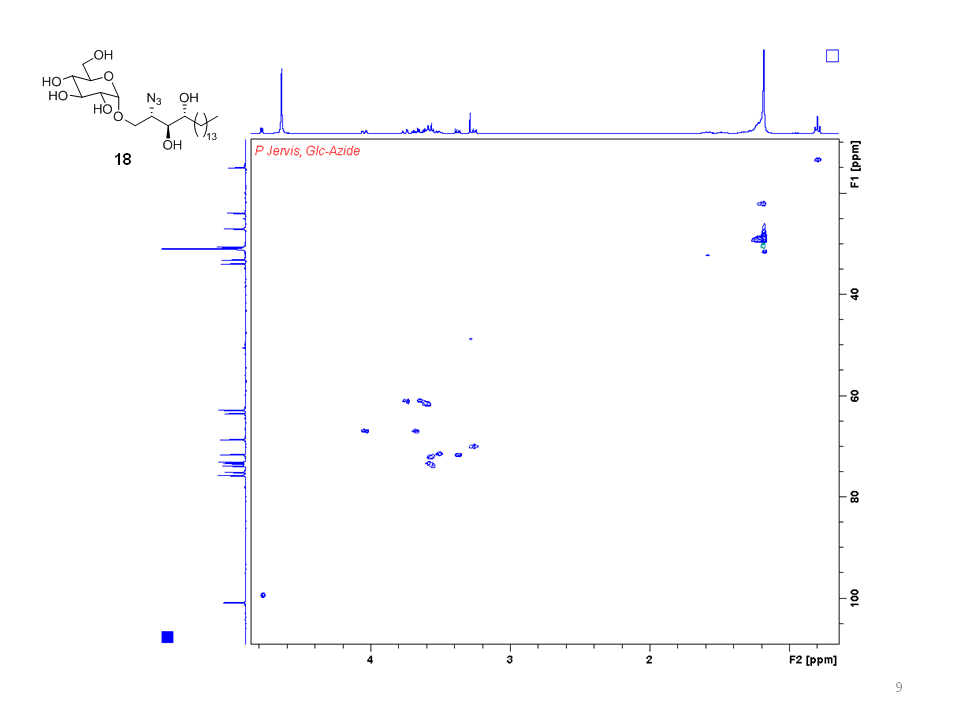

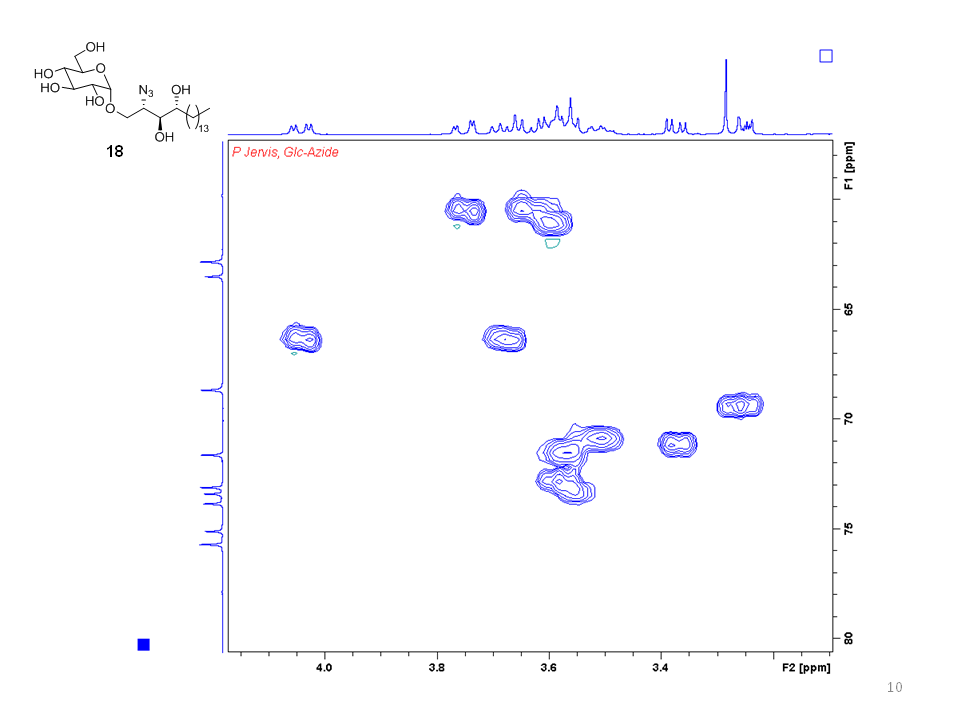

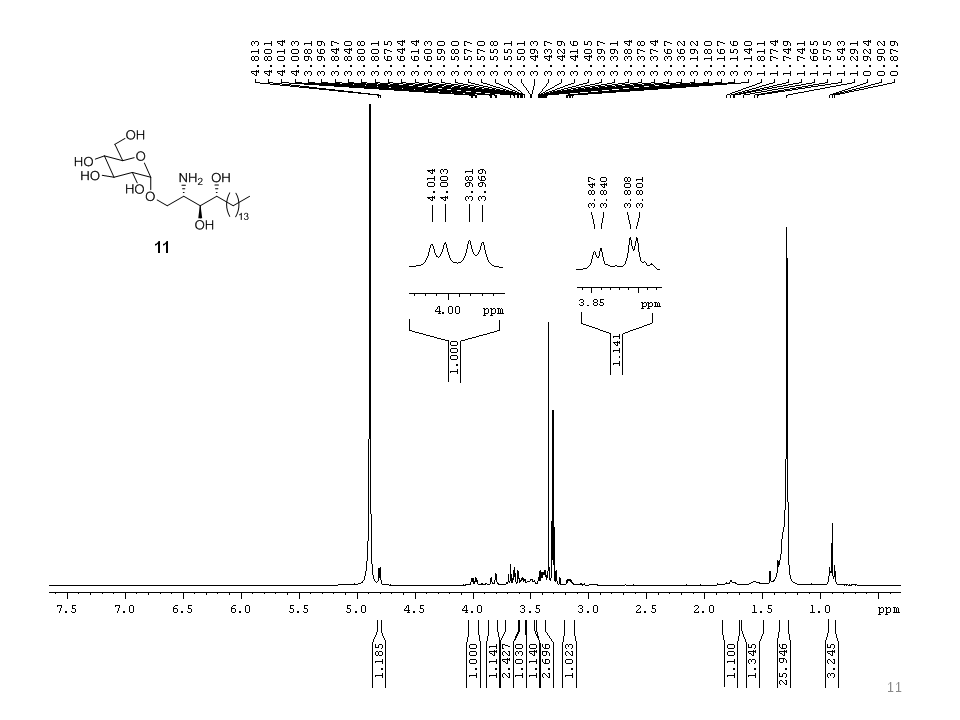

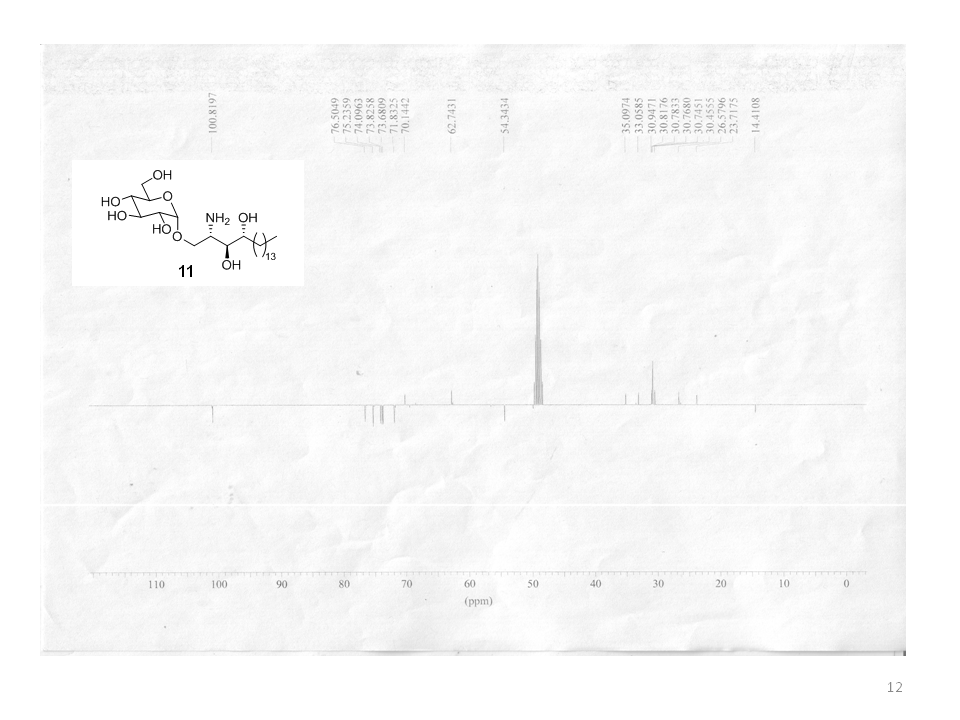

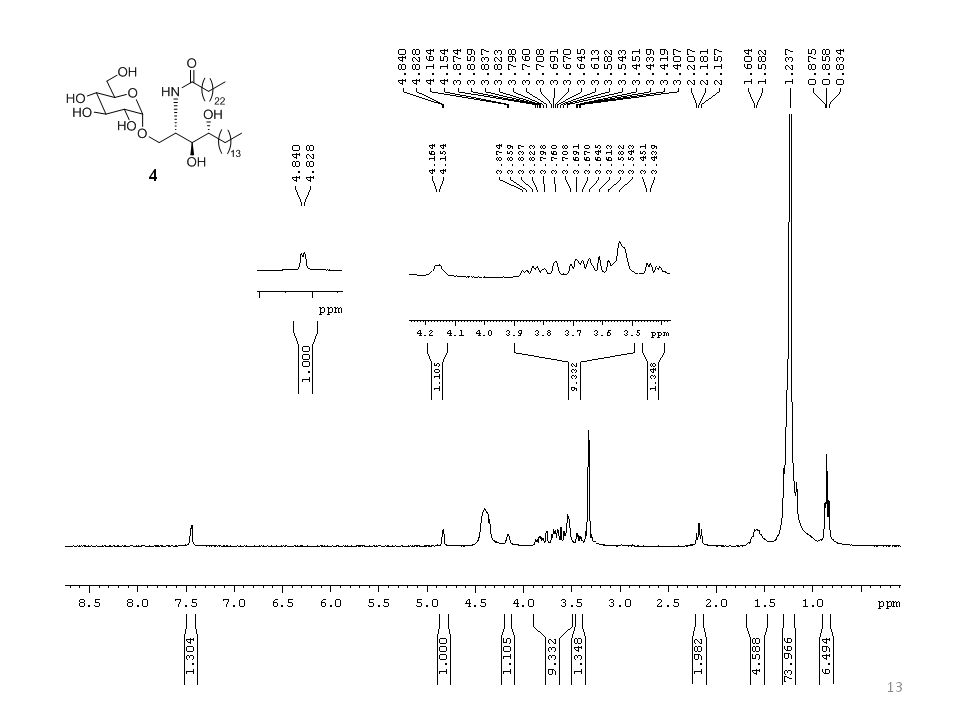

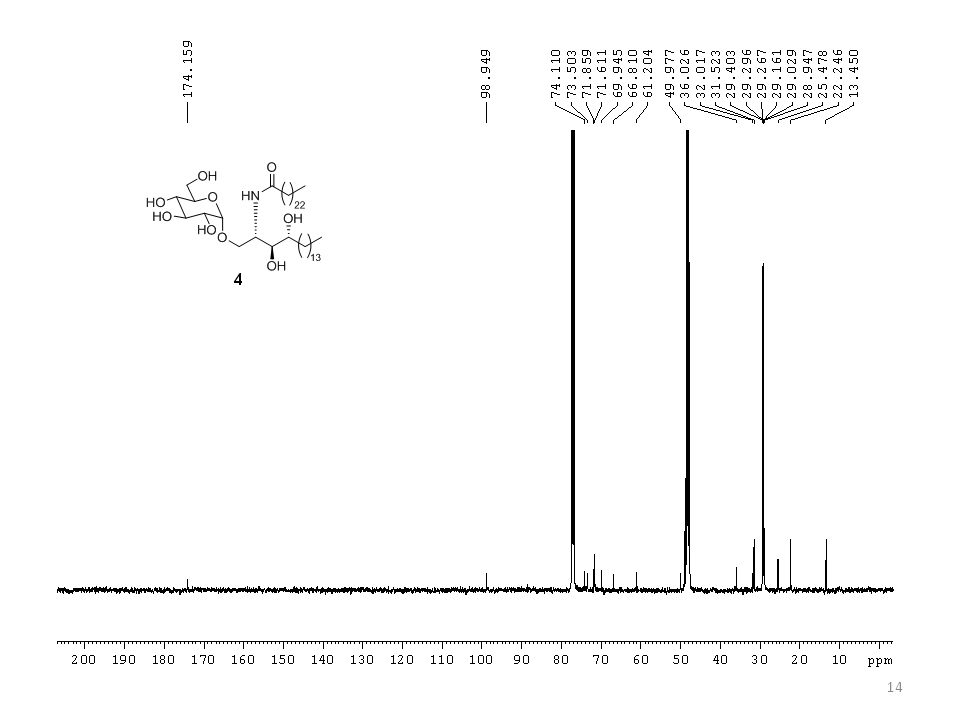

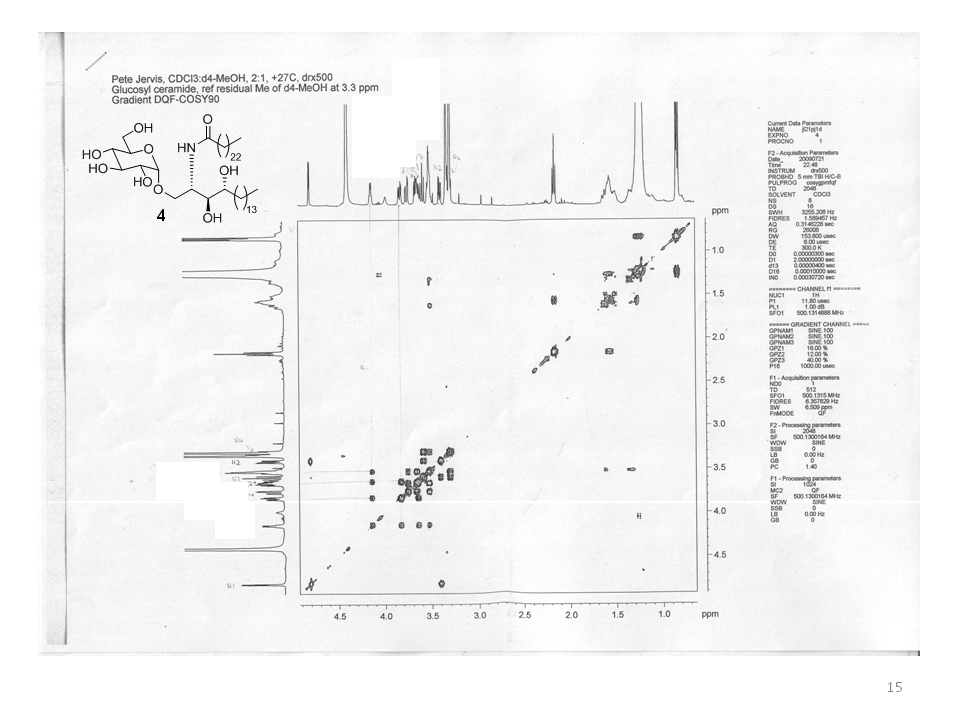

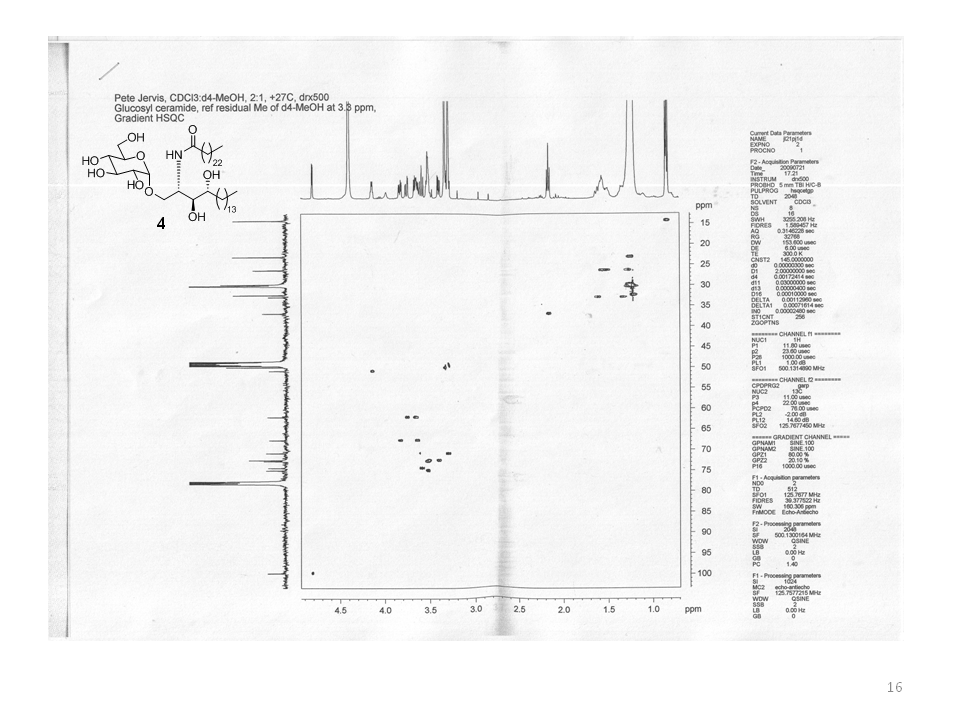

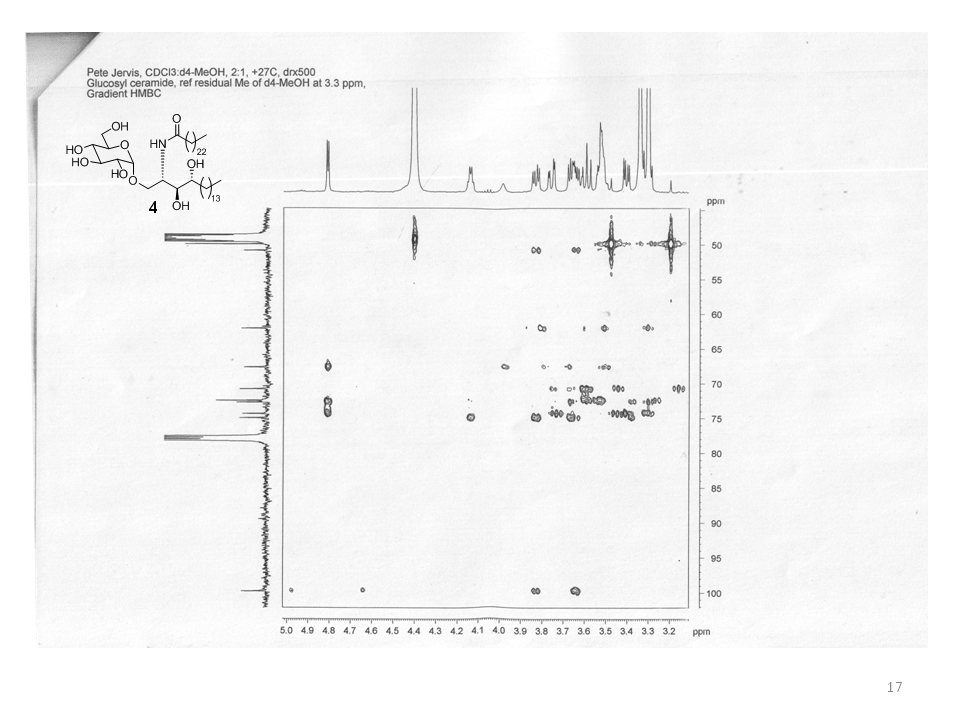

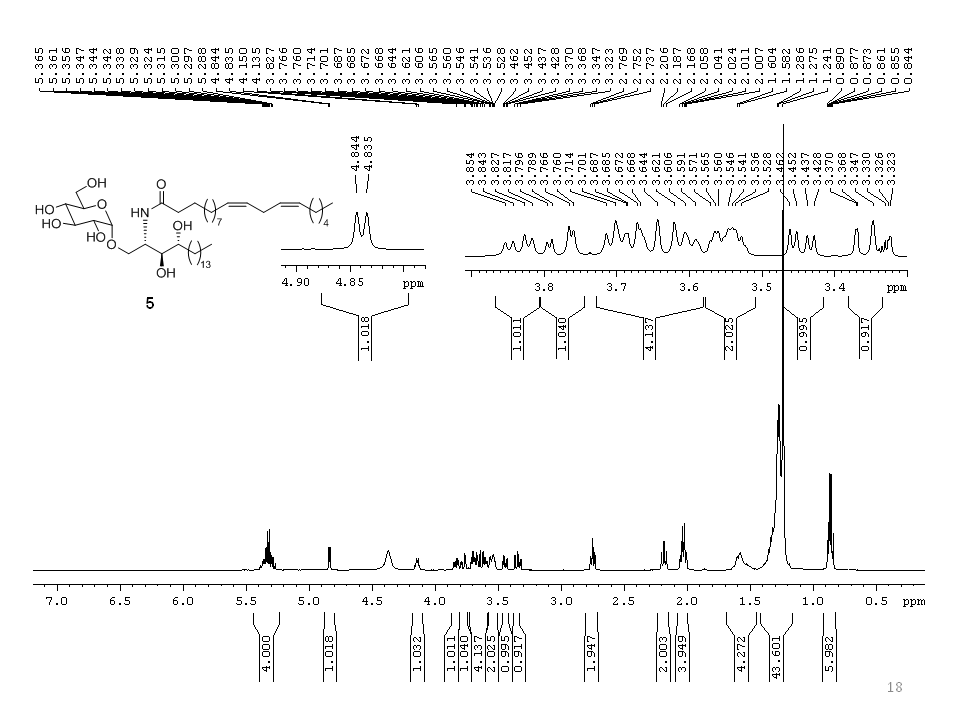

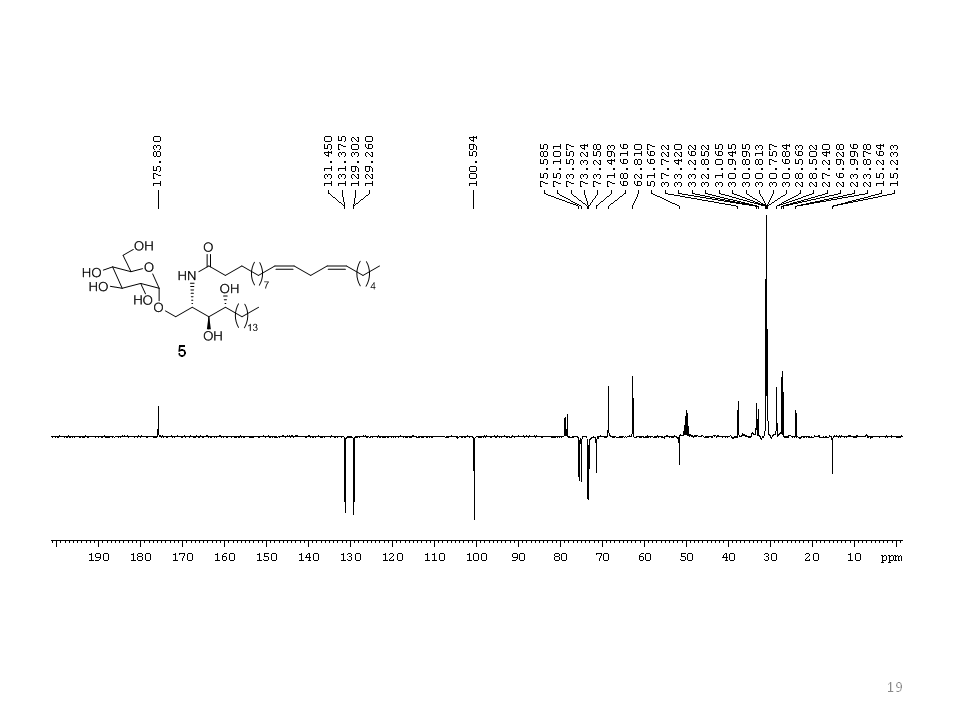

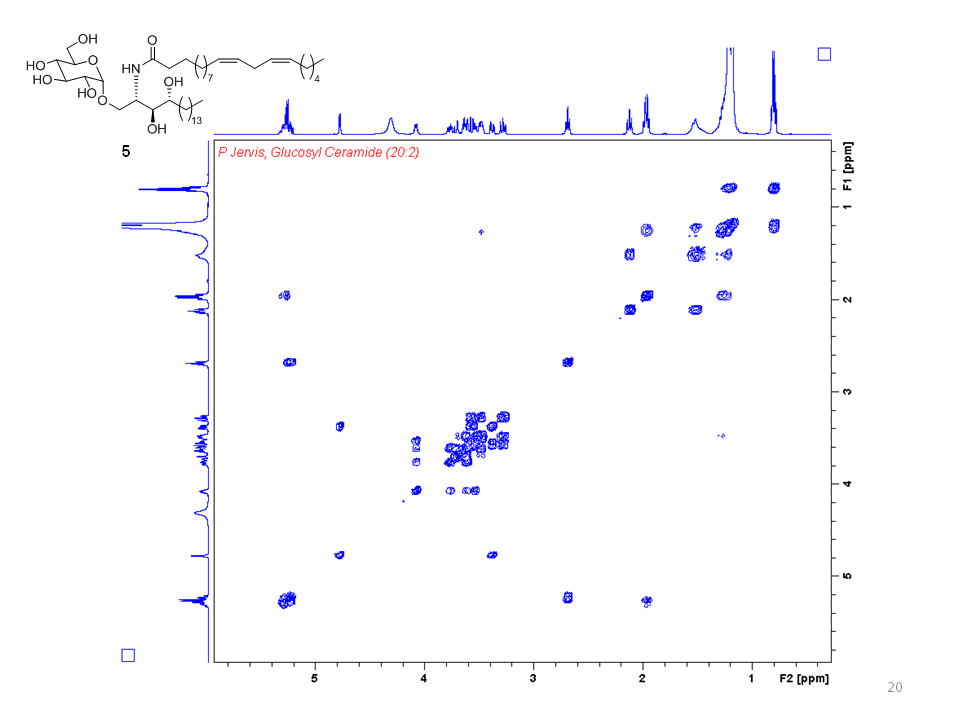

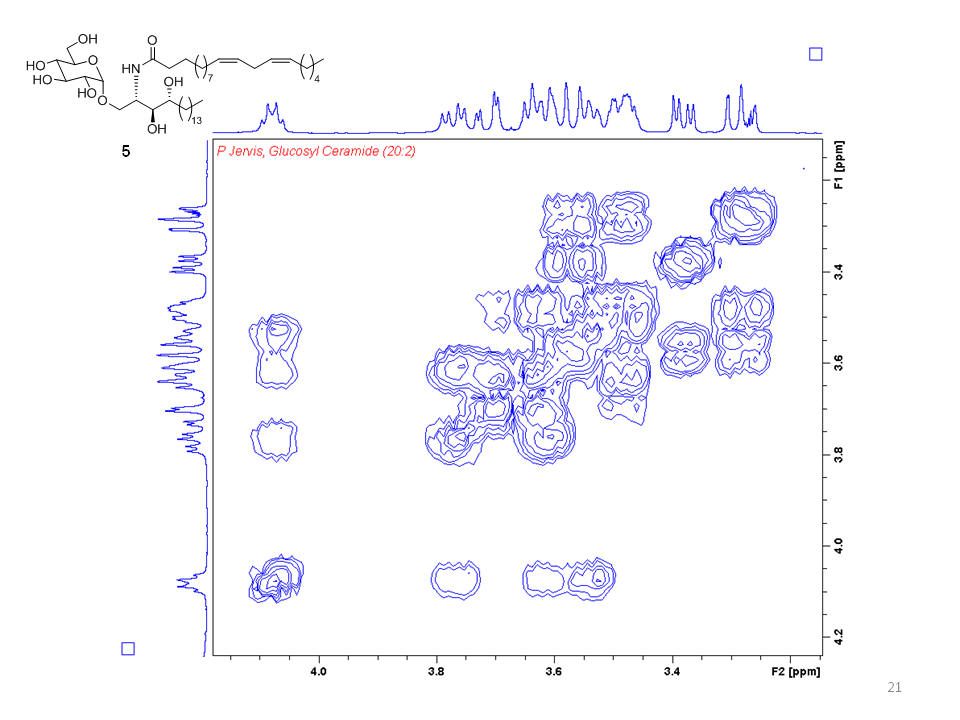

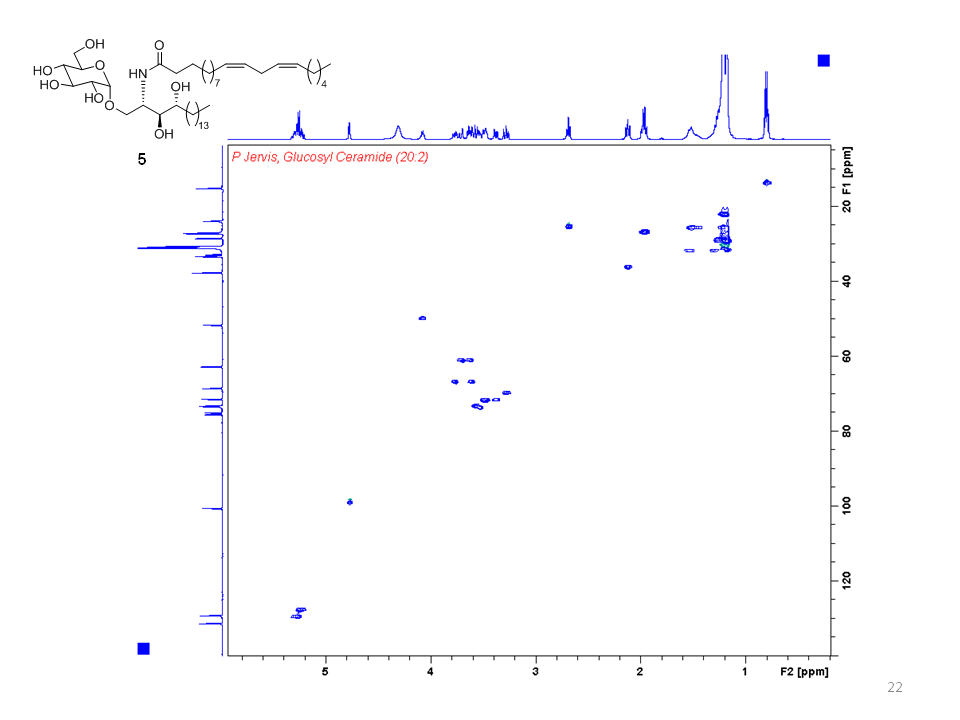

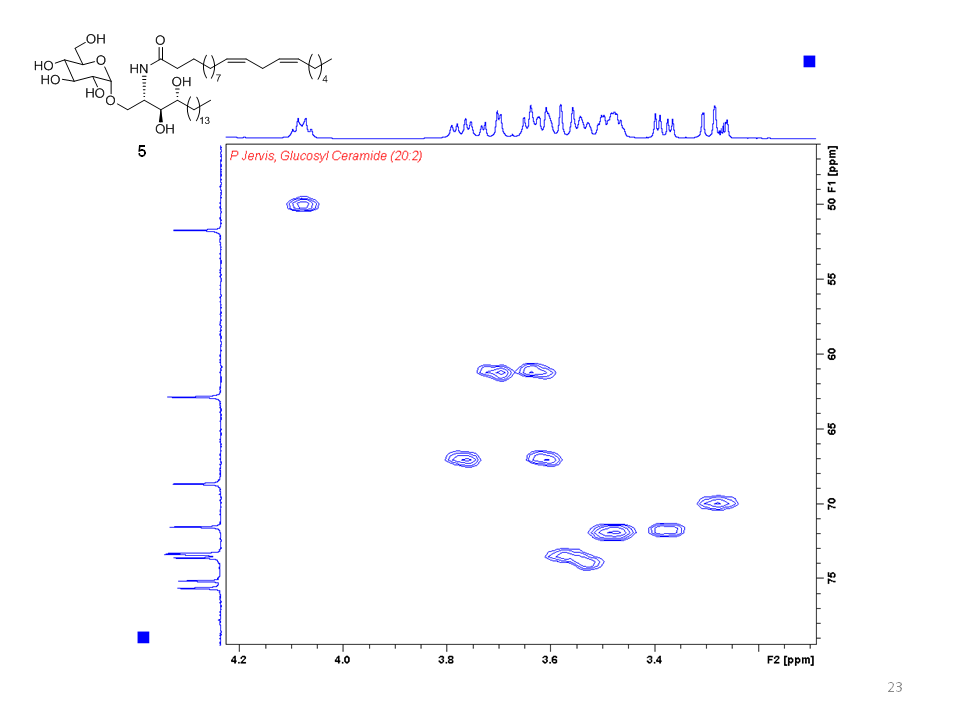


**18**
